# Targeting ErbB and tankyrase1/2 prevent the emergence of drug-tolerant persister cells in ALK-positive lung cancer

**DOI:** 10.1038/s41698-024-00757-w

**Published:** 2024-11-17

**Authors:** Takaaki Fujimura, Koh Furugaki, Hayato Mizuta, Satoshi Muraoka, Makoto Nishio, Jun Adachi, Ken Uchibori, Eisaku Miyauchi, Hidetoshi Hayashi, Ryohei Katayama, Shigeki Yoshiura

**Affiliations:** 1 Product Research Department, Chugai Pharmaceutical Co., Ltd, Yokohama, Japan; 2 Laboratory of Proteomics for Drug Discovery, Laboratory of Clinical and Analytical Chemistry, Center for Drug Design Research, National Institutes of Biomedical Innovation, Health and Nutrition, Osaka, Japan; 3 Department of Respiratory Medicine, The Cancer Institute Hospital of Japanese Foundation for Cancer Research, Tokyo, Japan; 4 Department of Respiratory Medicine, Tohoku University Graduate School of Medicine, Sendai, Japan; 5 Department of Medical Oncology, Kindai University Faculty of Medicine, Sayama, Japan; 6 Division of Experimental Chemotherapy, Cancer Chemotherapy Center, Japanese Foundation for Cancer Research, Tokyo, Japan

**Keywords:** Cancer therapeutic resistance, Targeted therapies, Non-small-cell lung cancer, Target identification

## Abstract

Targeting the drug tolerant persister (DTP) state in cancer cells should prevent further development of resistance mechanisms. This study explored combination therapies to inhibit alectinib-induced DTP cell formation from anaplastic lymphoma kinase–positive non-small cell lung cancer (ALK + NSCLC) patient–derived cells. After drug-screening 3114 compounds, pan-HER inhibitors (ErbB pathway) and tankyrase1/2 inhibitors (Wnt/β-catenin signaling) emerged as top candidates to inhibit alectinib-induced DTP cells growth. We confirmed knockdown of both TNKS1/2 in DTP cells recovered the sensitivity to alectinib. Further, our study suggested knockdown of TNKS1/2 increased stability of Axin1/2, which induced β-catenin degradation and decreased its nuclear translocation, thereby suppressing transcription of antiapoptotic and proliferation-related genes (*survivin*, *c-MYC*). Targeting both pathways with alectinib+pan-HER inhibitor and alectinib+TNKS1/2 inhibitor suppressed alectinib-induced DTP cells, and the triple combination almost completely prevented the appearance of DTP cells. In conclusion, combination with ALK-TKI, pan-HER and TNKS1/2 inhibitors has the potential to prevent the emergence of DTP in ALK + NSCLC.

## Introduction

The treatment outcome of patients with advanced non-small cell lung cancer (NSCLC) with anaplastic lymphoma kinase (ALK) chromosomal rearrangements, a distinct subset of lung cancer, has been improved by the development of tyrosine kinase inhibitors (TKIs)^1^. Several EML4-ALK fusion variants have been identified, with EML4-ALK being the dominant fusion variant, accounting for approximately 85% of all fusion variants in ALK + NSCLC^2^.

ALK inhibitors are effective and include ALK-TKIs such as crizotinib, alectinib, ceritinib, brigatinib, and lorlatinib. Among the TKIs, alectinib^3^ is a preferred first-line treatment based on its efficacy and side effect profile^4^ established through pivotal Phase 3 trials^4–7^.

Of note, resistance to TKIs is well known, and the development of resistant tumor cells is due to bypass/target modification mechanisms or modifications that cause phenotypic changes, target dependency, and loss of target^8^. These resistance mechanisms develop initially in a small population of tumor cells which achieve a slow, reversible, drug-tolerant persister (DTP) state^9^; over time, these cells acquire resistance to such TKIs through multiple additional mechanisms^10^. Therefore, targeting the DTP state with novel therapies should ideally be the first step in preventing further development of resistance mechanisms. Consequently, we believe that the direction of treatment will be to focus on multitarget therapy for both prevention and management of additional resistance mechanisms.

Numerous DTP appearance mechanisms have been described in the literature for ALK inhibitors^11–14^. For crizotinib, the mechanism was attributed to dynamic transcriptional responses—remodeling of enhancers in NSCLC-derived H3122 cells^11,12^. For alectinib, the mechanism has been attributed to transcriptional regulation by the activation of Yes-associated protein1 (YAP1; target for therapy: the YAP-TEAD pathway) in patient-derived NSCLC cell lines^10,13^. Both alectinib and brigatinib were shown to induce DTP appearance mechanisms by activating human epidermal growth factor receptor 3 (HER3) and through mesenchymal-to-epithelial transition in cell-based assays. When the ALK-TKI was combined with a pan-HER inhibitor, afatinib, it suppressed HER3 activation and promoted strong antitumor effect in ALK-rearranged tumors with mesenchymal features in a xenograft tumor model^14^.

Although alectinib remains the standard of care for ALK + NSCLC, some of the ALK+ lung cancer cells transiently become resistant to alectinib, and, over time, this leads to treatment resistance. However, an effective therapy to inhibit the residual DTP formation by alectinib in ALK + NSCLC has not yet been established. In this study, we explored novel dual and triple combination therapies for preventing the development of DTP cells using patient-derived ALK + NSCLC cells.

## Methods

### Cell lines

Patient-derived ALK1510 and ALK0413 cells were established from patients with ALK fusion gene–positive NSCLC who had provided informed consent for genetic and cell-biological analyses in the ALCURE (ALeCtinib Ultimate REsistance mechanisms) study (UMIN000038934). ALK1510 cells were established from a patient on the second day of alectinib treatment and ALK0413 cells were established from ALK-TKIs treatment naïve patient (Supplementary Table). EML4-ALK variant 1–expressing ALK1510-c4 cells were established by single-cell cloning from ALK1510 cells and maintained in equal proportions of RPMI-1640 medium (Sigma-Aldrich, St. Louis, MO, USA) and Ham’s F-12 medium (FUJIFILM Wako Pure Chemical Corporation, Osaka, Japan), and supplemented with 15% fetal bovine serum (Sigma-Aldrich) and 20 mM HEPES (NACALAI TESQUE Inc., Kyoto, Japan). EML4-ALK variant 2–expressing ALK0413 cells were maintained in StemPro hESC SFM (Thermo Fisher Scientific, Waltham, MA, USA) with 10 μM Y-27632 (AdooQ BioScience, Irvine, CA, USA) and cultured in a collagen-coated flask. Cells were maintained at 37 °C under 5% CO_2_. A549 cells (ATCC, Manassas, VA, USA) were maintained in F-12K (Thermo Fisher Scientific) supplemented with 10% fetal bovine serum.

### Drugs and reagents

Alectinib was synthesized by Chugai Pharmaceutical Co., Ltd. (Tokyo, Japan). For in vitro assays, lorlatinib, dacomitinib, AZ6102, G007-LK, XAV939, LY2090314, AR-A014418, and omecamtiv mecarbil were purchased from Selleck Chemicals (Houston, TX, USA). Neratinib was obtained from LC Laboratories (Woburn, MA, USA). All agents were dissolved in dimethyl sulfoxide (DMSO; Sigma-Aldrich) for in vitro assays. For compound library screening, anticancer compound library was obtained from TargetMol (Boston, MA, USA). For in vivo assays, dacomitinib was purchased from TargetMol and G007-LK from ChemScene (Monmouth Junction, NJ, USA). Both reagents were dissolved in 5% DMSO v/v, 30% PEG 300 v/v (FUJIFILM Wako Pure Chemical Corporation), and 5% Tween 80 v/v (Sigma-Aldrich). Alectinib was dissolved in 6% (w/v) solution of Captisol® (ChemScene).

### Cell proliferation assay

Cells were seeded in 384-well plates and drugs were added at the indicated concentrations (see figure legends) on the following day. After 6 days, cell viability was determined using the CellTiter-Glo™ 3D Cell Viability Assay (Promega, Madison, WI, USA). Viability with the agents relative to viability with an untreated control was measured. Each data point shown in the results section represents the mean (standard deviation [SD]) of triplicate experiments.

For compound library screening, ALK1510-c4 cells and ALK1510-c4 DTP cells were seeded in 384-well plates and 3114 agents of an anticancer compound library were added at 100 nM on the following day. After 6 days, cell viability was determined using the CellTiter-Glo™ 3D Cell Viability Assay. Viability with the agents relative to viability with the untreated control was measured, and the antiproliferative effect of each agent on ALK1510-c4 DTP cells was calculated as follows: viability of DTP cells/viability of parental cells.

### Western blotting

Cells were seeded in 6-well plates and drugs were added at the indicated concentrations on the following day and cultured for the indicated time (see respective figure legends). The same amount of protein lysate was loaded for each western blotting assay using the Sally Sue™ or Jess™ capillary electrophoresis–based protein analysis system (ProteinSimple, Santa Clara, CA, USA) per the manufacturer’s protocol. Antibodies against ALK, phosphorylated ALK, extracellular signal-regulated kinase (ERK), phosphorylated ERK (pERK), protein kinase B (AKT), phosphorylated AKT (pAKT), signal transducer and activator of transcription 3 (STAT3), phosphorylated STAT3 (pSTAT3), epidermal growth factor receptor (EGFR), phosphorylated-EGFR, β-actin, human epidermal growth factor receptor 2 (HER2), HER3, phosphorylated HER3 (pHER3), β-catenin, phosphorylated-β-catenin, c-MYC, survivin, Bcl-2-like protein 11 (BIM), vimentin, E-cadherin, cluster of differentiation 133 (CD133), β-tubulin, NDRG1 (Cell Signaling Technology, Danvers, MA, USA), EGF, NRG2, cleaved poly (ADP-ribose) polymerase (PARP), lamin A/C, Axin2 (Abcam), and Axin1 (Thermo Fisher Scientific) were used. Each primary antibody was diluted 25 to 1000 fold.

### Phosphoproteomic analysis

After ALK1510-c4 cells were treated with 1000 nM alectinib for 3 h or 9 days (DTP), cells were washed with PBS supplemented with PhosSTOP (Roche Diagnostics, Basel, Switzerland) and cOmplete EDTA-free (Roche Diagnostics) and collected with scrapers. After centrifugation, cell pellets were frozen in liquid nitrogen and stored at −80 °C until usage. A total of 2 mg of each sample in PTS buffer was reduced with 10 mM TCEP, alkylated with 20 mM iodoacetamide, and quenched with 21 mM of L-cysteine. Samples were digested with trypsin (protein weight: 1/50) and Lys-C (protein weight: 1/50) for 16 h at 37 °C. Samples were acidified with 1% TFA and centrifuged at 20,000 g for 10 min at 4 °C. Supernatants were desalted and applied IMAC column for phosphopeptide enrichment^15^. TMT 10plex reagents (0.8 mg, Thermo Scientific, Bremen, Germany) were dissolved in anhydrous acetonitrile (45 μL) of which 4 μL was added to the phosphopeptides desolved with 10 μL of 100 mM TEAB. Following incubation at room temperature for 1 h, the reaction was quenched with hydroxylamine for a final concentration of 0.3% (v/v). The TMT-labeled samples were pooled and divided into 28.6% for global phosphoproteome analysis and 71.6% for phosphotyrosine proteome analysis. Both samples were vacuum centrifuged to near dryness. For global phosphoproteome analysis, TMT-labeled phosphopeptides were subjected to off-line basic pH reversed-phase (BPRP) fractionation. We fractionated the pooled TMT-labeled peptide sample using BPRP HPLC. We used a Thermo Scientific UltiMate 3000 UHPLC system equipped with a dual wavelength detector (set at 220 nm and 280 nm). The mobile phases were BPRP-A (5 mM ammonium bicarbonate pH 9.2) and BPRP-B (5 mM ammonium bicarbonate pH 9.2 and 60% acetonitrile). The LC gradient was carried out as follows: 5–25% BPRP-B for 4 min, 25%–60% BPRP-B for 26 min and then 60–99% BPRP-B for 15 min. TMT-labeled phosphopeptides were separated by L-column3 C18 column (5 µm particles, 0.3 mm ID and 150 mm in length, Chemicals Evaluation and Research Institute, Tokyo, Japan) at the flow rate of 2 µL/min. The peptide mixture was fractionated into a total of 21 fractions which were consolidated into 7 fractions. Samples were subsequently vacuum centrifuged to near dryness. Phosphotyrosine enrichment was performed using pY1000 antibody as previously reported^16^. Each fraction was reconstituted in 2% acetonitrile, 0.1% trifluoroacetic acid for LC-MS/MS processing.

LC-MS/MS was performed by coupling an UltiMate 3000 Nano LC system (Thermo Scientific) and an HTC-PAL autosampler (CTC Analytics, Zwingen, Switzerland) to an Orbitrap Fusion Lumos mass spectrometer (Thermo Scientific). Peptides were delivered to an analytical column (75 μm × 30 cm, packed in-house with ReproSil-Pur C18-AQ, 1.9 μm resin, Dr. Maisch, Ammerbuch, Germany) and separated at a flow rate of 280 nL/min using a 145-min gradient from 5% to 30% of solvent B (solvent A, 0.1% FA; solvent B, 0.1% FA and 99.9% acetonitrile). The Orbitrap Fusion Lumos mass spectrometer was operated in the data-dependent mode. For global phosphoproteome analysis, survey full scan MS spectra (*m/z* 375 to 1500) were acquired in the Orbitrap with 120,000 resolution after accumulation of ions to a 4 × 10^5^ target value. Maximum injection time was set to 50 ms and dynamic exclusion was set to 30 s. MS2 analysis consisted of higher-energy collisional dissociation (HCD); AGC 1 × 10^5^; normalized collision energy (NCE) 38; maximum injection time 105 ms; 50,000 resolution and isolation window of 0.7 Da. For phosphotyrosine proteome analysis, survey full scan MS spectra (*m/z* 350 to 1500) were acquired in the Orbitrap with 120,000 resolution after accumulation of ions to a 4 × 10^5^ target value. Maximum injection time was set to 100 ms and dynamic exclusion was set to 5 s. MS2 analysis consisted of higher-energy collisional dissociation (HCD); AGC 1 × 10^5^; normalized collision energy (NCE) 38; maximum injection time 315 ms; 120,000 resolution and isolation window of 0.7 Da.

Raw MS data were processed by MaxQuant (version 1.6.14.0) supported by the Andromeda search engine. The MS/MS spectra were searched for within the UniProt human database using the following search parameters: full tryptic specificity, up to two missed cleavage sites, carbamidomethylation of cysteine residues set as a fixed modification, and serine, threonine, and tyrosine phosphorylation, N-terminal protein acetylation and methionine oxidation as variable modifications. The false discovery rate (FDR) of protein groups, peptides, and phosphosites were less than 0.01. Quantitative values of the phosphorylation sites across different fractions were automatically integrated and summarized in “Phospho (STY) Sites.txt” by MaxQuant. Peptides that were identified from the reversed database, or identified as potential contaminants were not used in the following analysis. Kinase activity prediction was performed using site-centric posttranslational modification-signature enrichment analysis (PTM-SEA)^17^, a seven-amino-acid sequence flanking the phosphosite as an identifier and the human kinase/pathway definitions of PTMsigDB (v.2.0.0) according to the following parameters: (gene.set.database = “ptm.sig.db.all.flanking.human.v2.0.0.gmt”, sample.norm.type = “rank”, weight = 0.75, statistic = “area.under. RES”, output.score.type = ”NES”, nperm = 1000, global.fdr = TRUE, min.overlap = 3, correl.type = “z.score”).

### Preparation of nuclear extracts

Cells were harvested using a cell scraper, and the cell lysates were divided into cytosolic and nuclear fractions using the Nuclear/Cytosolic Fractionation Kit (Cell Biolabs, Inc., CA, USA) per the manufacturer’s protocol. Cell extracts were subjected to western blotting as described above.

### ELISA

Cells were seeded in a 10-cm dish, and the following day, drugs were added at the indicated concentrations and the cells cultured for the indicated time (see respective figure legends). The same amount of protein lysate was loaded for each enzyme-linked immunosorbent assay (ELISA) conducted using the PathScan® phospho-HER2/erythroblastic oncogene B-2 (ErbB2) (panTyr) Sandwich ELISA Kit (Cell Signaling Technology) according to the manufacturer’s protocol.

### RNA-seq analysis

For RNAseq analysis of ALK1510-c4 cells, total RNA was obtained from ALK1510-c4 or its DTP cells with or without 1000 nM alectinib treatment for 24 h and 9 days using the Maxwell RSC simplyRNA Tissue Kit (Promega). Transcriptome libraries were prepared using the TruSeq Stranded mRNA Library Preparation Kit (Illumina, San Diego, CA, USA), and next-generation sequencing was performed on a NovaSeq6000 system (Illumina) with paired-end 150 bp reads at Macrogen Japan Corp (Tokyo, Japan). After excluding read pairs with a mapping quality of <20 using Trimmomatic, the sequence reads were aligned to the Hg38 reference genome using STAR, and read count data was obtained with prepDE.py.

### RT-PCR assay

mRNA was purified using the Maxwell RSC instrument (Promega), and complementary DNA (cDNA) was synthesized using the PrimeScript RT reagent kit (Takara Bio Inc., Shiga, Japan). Real-time polymerase chain reaction (RT-PCR) was performed using the LightCycler® 480 Instrument II (Roche Diagnostics, Basel, Switzerland) and TaqMan probes targeting human tankyrase 1 (TNKS1; Hs00186671_m1), human TNKS2 (Hs00961183_m1), and human glyceraldehyde 3-phosphate dehydrogenase (GAPDH; 4352665).

### Caspase-3/7 assay

Cells were seeded in 384-well plates, and the following day, drugs were added at the indicated concentrations and the cells cultured for 4 days. The activity of caspases 3 and 7 was determined using the Caspase-Glo® 3/7 Assay (Promega). The activity was corrected with respect to the cell viability determined as described above, and the relative activity with the agents against untreated control was calculated. Each data point represents the mean (SD) of triplicate experiments.

### Transfection of siRNA

Cells were transfected with ON-TARGETplus predesigned small interfering RNA (siRNA) SMARTpool targeting EGFR, ErbB2, ErbB3, TNKS1, TNKS2, CTNNB1 or nontarget control (Horizon Discovery, Cambridge, UK) using lipofectamine RNAiMax transfection reagent (Thermo Fisher Scientific) according to the manufacturer’s protocol. After 2 days, cells were harvested and reseeded for each assay; the cells were treated with drugs on the following day at the indicated concentration in combination with siRNAs (see respective figure legends).

### Animals

Animal procedures were approved by the Institutional Animal Care and Use Committee at Chugai Pharmaceutical Co., Ltd. (IACUC) and conformed to the Guide for the Care and Use of Laboratory Animals published by Institution of Laboratory Animal Research (ILAR). The IACUC approved the animal experiments with the approval number 22-015. Five-week-old male severe combined immunodeficiency (SCID) mice (C.B-17/Icr-scid/scidJcl) were purchased from CLEA Japan, Inc. (Tokyo, Japan). All animals were acclimatized for >5 days prior to the study. Chlorinated water and irradiated food were provided ad libitum, and the animals were kept under 20–26 °C temperature, 30–60% humidity, and a controlled 12–12 h light-dark cycle.

### Long-term in vitro efficacy

Cells were seeded in six 25-cm^2^ flasks per group and treated with drugs at the indicated concentration on the following day (see respective figure legends). From Week 1 to Week 5, cells were collected from one flask per group, suspended in 1 mL of the medium, and counted using the NC-3000 Advanced Image Cytometer (ChemoMetec, Allerod, Denmark). The remaining flasks had a culture medium change containing each drug each week. From Week 6 onward, the flasks had a culture medium change without any drug each week, and cells were counted at Week 11.

### Mouse xenograft models

Mice were inoculated subcutaneously with 5 × 10^6^ cells per mouse into the right flank. The tumor volume and body weight were measured twice a week, and the tumor volume was estimated as follows: tumor volume = ab^2^/2, where a and b are tumor length and width using the Chugai Antitumour Evaluation System (ANTES)^18^ respectively. After tumor establishment, mice were openly and randomly allocated to a 14-day treatment with the vehicle or indicated drug or drug combination with ANTES system, wherein each compound was orally administered daily at the same dose and schedule as the single agent. The oral administration route for each drug was selected based on previously published studies^19–21^. In compliance with IACUC policies, the study was conducted with predefined humane endpoints, which included tumor size exceeding 10% of body weight or rapid weight loss of more than 20% within a week as criteria for euthanasia, and mice were euthanized by cervical dislocation under anesthesia with isoflurane inhalation, or by carbon dioxide.

### Statistical analysis

In vitro experimental data were analyzed using the Student’s *t* test, Tukey’s HSD test or Dunnett’s test. Two-way ANOVA was used prior to each test for comparison of cell proliferation assay. The in vivo experimental data were analyzed using the Wilcoxon rank sum test, followed by the Holm–Bonferroni method. All statistical analyses were conducted using JMP Ver. 15.0.0 (SAS Institute, Cary, NC, USA). Significance was set at two-tailed *P* < 0.05.

## Results

### Preparation and characterization of ALK1510-c4 DTP cells from ALK1510-c4 cells

**Fig. 1:**
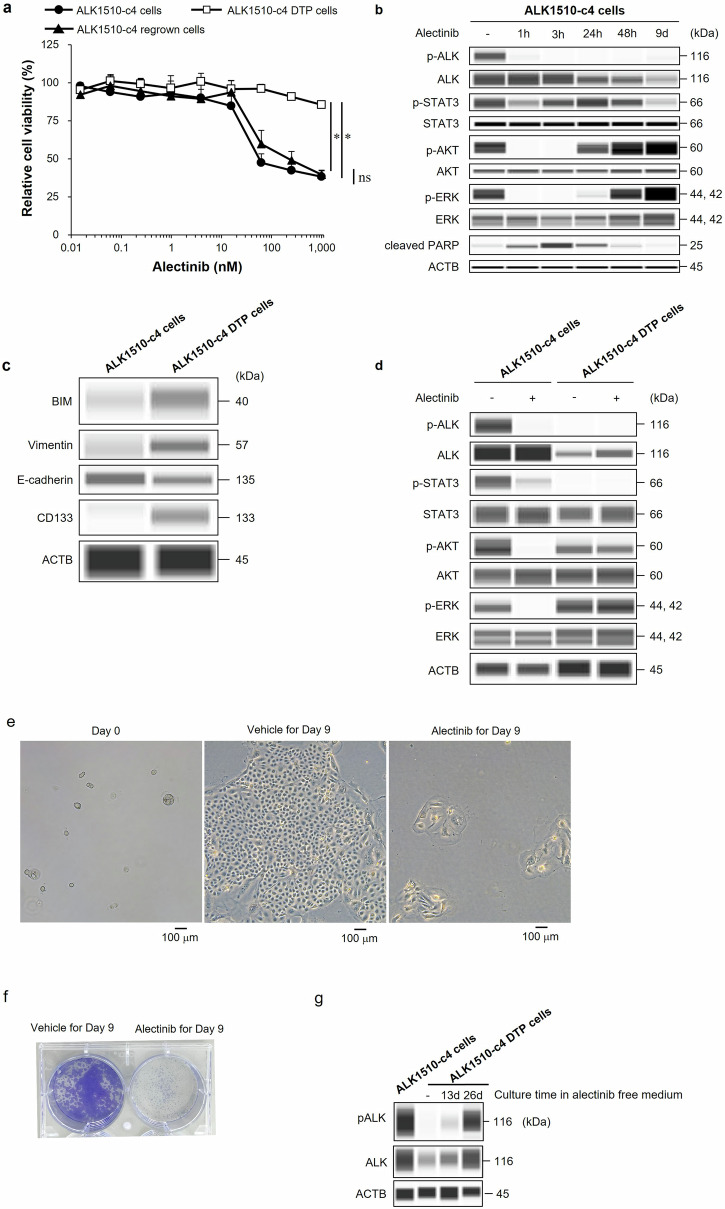
Preparation and characterization of ALK1510-c4 DTP cells from ALK1510-c4 cells. **a** ALK1510-c4 DTP cells were generated from ALK1510-c4 cells after treatment with 1000 nM alectinib for 9 days, ALK1510-c4 regrown cells were generated from ALK1510-c4 DTP cells cultured in alectinib-free cell culture for 37 days, and comparison with ALK1510-c4 cell controls were assessed in a cell proliferation assay (mean [SD] of *n* = 3 experiments, *P < 0.05 between ALK1510-c4 and ALK1510-c4 DTP and ALK1510-c4 regrown; Tukey’s HSD test). **b** Immunoblots of cell lysates showing changes in phosphorylated and/or total ALK, AKT, ERK, STAT3, and cleaved PARP in ALK1510-c4 cells treated with 1000 nM alectinib for 1, 3, 24, and 48 h and 9 days. **c** Immunoblots of cell lysates showing changes in BIM, stem cell marker CD133, vimentin, and E-cadherin protein levels in ALK1510-c4 cells and ALK1510-c4 DTP cells. **d** Immunoblots of cell lysates showing change in phosphorylated and/or total ALK, AKT, ERK and STAT3 protein levels in ALK1510-c4 cells and ALK1510-c4 DTP cells after treatment with 1000 nM alectinib for 1 h. Microscopic examination of ALK1510-c4 parental and DTP cells generated from ALK1510-c4 by treatment with 1000 nM alectinib for 9 days with phase contrast (**e**), and crystal violet staining (**f**). **g** Immunoblots of cell lysates in ALK1510-c4 DTP cells after 13 and 26 days culture in the alectinib-free medium.

The cell proliferation assay showed alectinib exerts potent activity in ALK1510-c4 cells established from an ALK+ lung cancer patient. Previous research showed that the DTP state caused by drug exposure is reversible^9^. Thus, to establish alectinib-induced ALK1510-c4 DTP cells, we treated ALK1510-c4 cells with 1000 nM alectinib for 9 days. While the established DTP cells showed at reduced inhibitory effect of alectinib, ALK1510-c4 DTP cells cultured in the absence of alectinib for 37 days (ALK1510-c4 regrown cells) resensitized to alectinib in a concentration-dependent manner (10 nM to 100 nM concentration), suggesting that the high sensitivity to alectinib had been restored (Fig. 1a).

Analysis of the time-dependence of ALK signal in ALK1510-c4 cells after exposure to 1000 nM alectinib showed that phosphorylation of ALK was completely inhibited from 1 h onward (for all timepoints assessed). The phosphorylation of ALK downstream signaling molecules, AKT and ERK, was inhibited at 1 and 3 h post-exposure and restored after 24 h. Cleaved PARP abundance, a marker of apoptosis, was elevated at 1 and 3 h post-exposure and declined after 24 h (Fig. 1b). Alectinib-induced ALK1510-c4 DTP cells showed increased expression of BIM, a pro-apoptotic B-cell lymphoma-2 (BCL2)-family protein; CD133, a stem cell marker; and vimentin, an epithelial mesenchymal transition (EMT) marker, and decreased expression of E-cadherin, an EMT marker (Fig. 1c). Compared with ALK1510-c4 cells, where the phosphorylation of ALK, AKT, ERK and STAT3 was inhibited at 1 h after alectinib exposure, the ALK1510-c4 DTP cells showed an absence of phosphorylated ALK. In addition, while phosphorylated STAT3 was not detected, our WB result showed the absence of inhibition of phosphorylated AKT and ERK at 1 h after alectinib exposure (Fig. 1d). Consistent with WB results, phosphoproteomics analysis showed suppression of pALK in alectinib 3 h and 9 days (DTP) treated ALK1510-c4 cells compared to vehicle (Supplementary Fig. 1a, b). Also, the phosphorylation of ERK was increased in DTP cells compared to alectinib 3 h treated cells (Supplementary Fig. 1c). Furthermore, ALK1510-c4 DTP cells showed features common to DTP cells, such as morphological alterations (Fig. 1e), and a small fraction of ALK1510-c4 cells survived and evolved as DTP cells after 9-day treatment with alectinib (Fig. 1e, f). ALK phosphorylation in ALK1510-c4 DTP cells was restored to similar levels as in parental ALK1510-c4 cells after 26 days culture in alectinib-free medium (Fig. 1g).

### Screening the compound library to identify drugs inhibiting the growth of ALK1510-c4 DTP cells

**Fig. 2:**
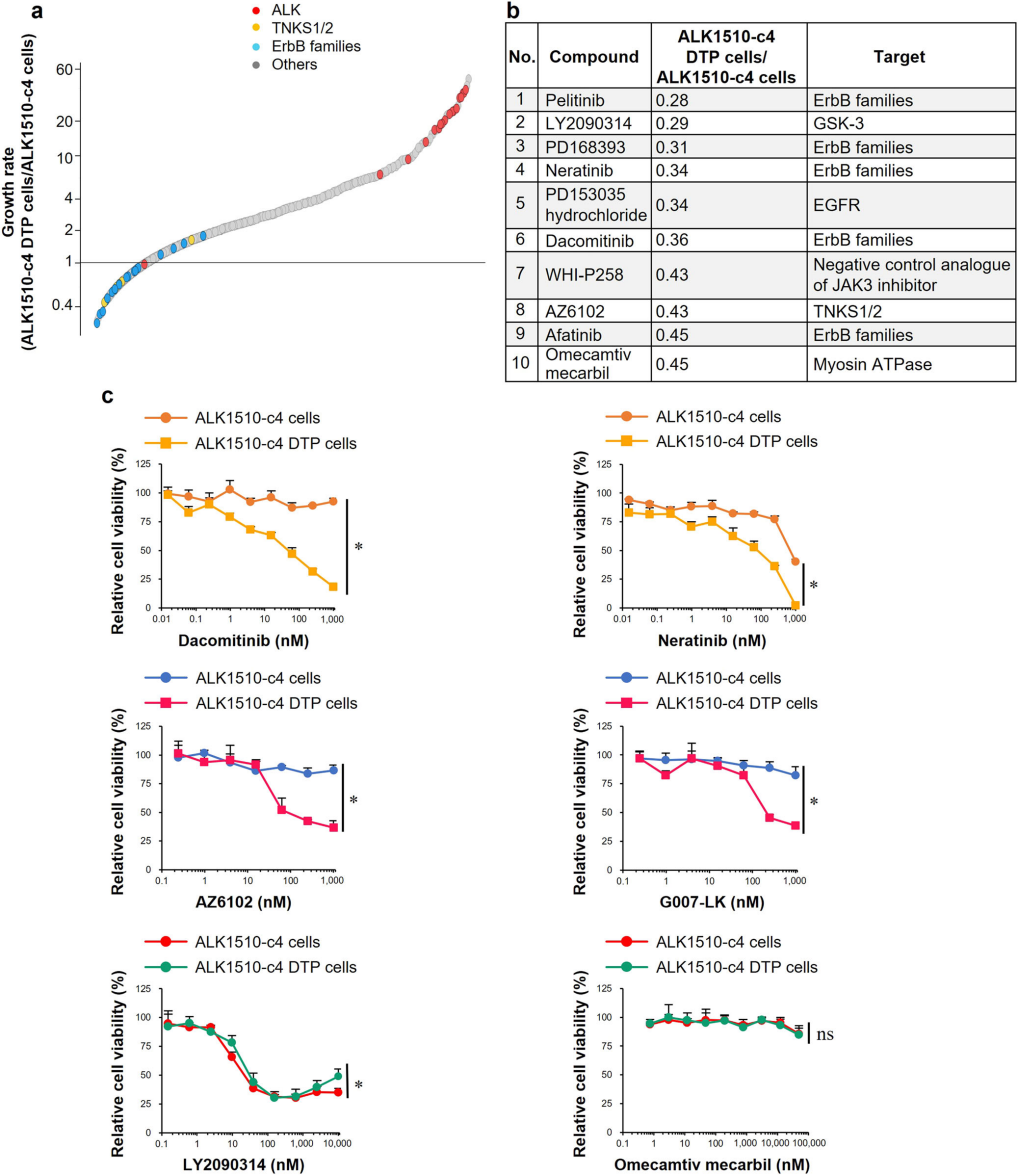
Screening the compound library to identify drugs with specific growth rate (ALK1510-c4 DTP cells/ALK1510-c4 cells). **a** ALK1510-c4 cells and ALK1510-c4 DTP cells were cultured with each of the 3114 compounds in an anticancer drug compound library (*n* = 1). Antiproliferative effects on ALK1510-c4 DTP cells relative to ALK1510-c4 cells are plotted as ratios in an ascending order for each compound by evaluating cell viability of the respective cells. **b** Top ten compounds with antiproliferative effects on ALK1510-c4 DTP cells relative to ALK1510-c4 cells are tabulated. **c** Cell proliferation assay/cytostatic studies showing relative cell viability/difference in drug sensitivity of ALK1510-c4 cells and ALK1510-c4 DTP cells after treatment with dacomitinib, neratinib, AZ6102, G007-LK, LY2090314, and omecamtiv mecarbil; mean [SD] of *n* = 3 experiments, *P < 0.05 between ALK1510-c4 and ALK1510-c4 DTP ; Student’s *t* test).

To identify targets that promote the growth of DTP cells, we compared growth inhibition between ALK1510-c4 DTP cells and ALK1510-c4 cells under drug exposure. Figure 2a shows compound-specific growth rates (ALK1510-c4 DTP cells/ALK1510-c4 cells) plotted in an ascending order using a 3114-anticancer drug library. The top 10 compounds that inhibited cell proliferation in ALK1510-c4 DTP cells (i.e., with a lower proliferation ratio) are shown in Fig. 2b (ratios in the range of 0.28–0.45). The targets of compounds included the ErbB family, GSK-3, TNKS1/2, and myosin ATPase activator, and indirectly represented potential sites. As WHI-P258 is a negative control analog of the Janus kinase 3 (JAK3) inhibitor, it was not included for further evaluation (Fig. 2b). The identified compounds or their mechanisms of action were validated using cytostatic studies (G007-LK, another TNKS1/2 inhibitor, was also evaluated). Dacomitinib and neratinib, pan-HER inhibitors of the ErbB family pathway, and AZ6102 and G007-LK, TNKS1/2 inhibitors, suppressed cell growth in ALK1510-c4 DTP cells compared with ALK1510-c4 cells. The cytostatic efficacy of LY2090314, a GSK-3 inhibitor, and omecamtiv mecarbil, a myosin ATPase activator, was almost unchanged in both ALK1510-c4 DTP cells and ALK1510-c4 cells, which means the reproducibility of drug screening was not observed in these drugs (Fig. 2c). These findings suggest that ALK1510-c4 cells are protected from alectinib-induced cell death through DTP mechanisms associated with the activation of cell growth signals through the ErbB pathway and/or TNKS1/2.

### Evaluation of ErbB signaling in alectinib-induced ALK1510-c4 DTP cells treated with the pan-HER inhibitor dacomitinib

**Fig. 3:**
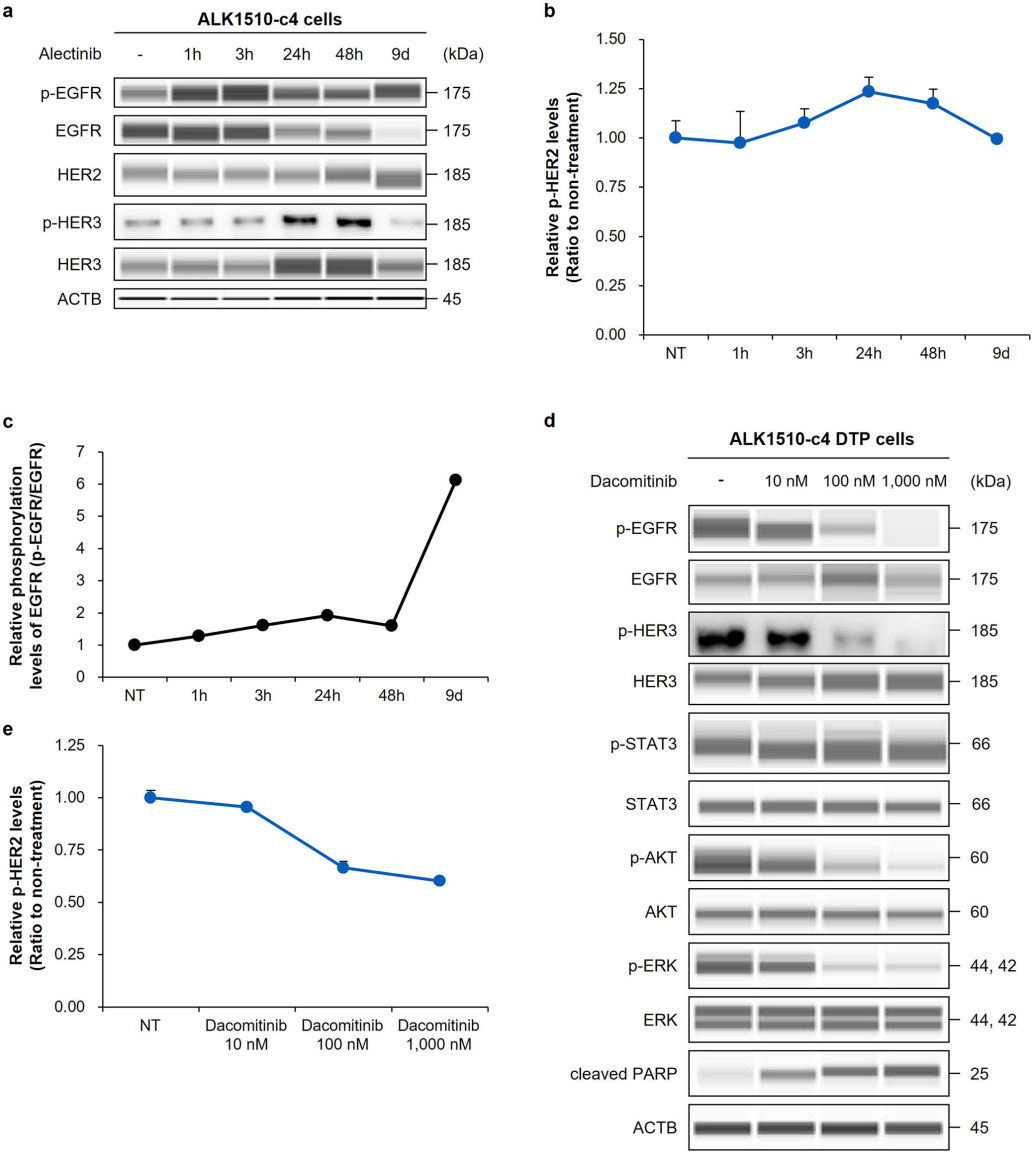
Evaluation of ErbB signaling in ALK1510-c4 cells using alectinib and ALK1510-c4 DTP cells treated with pan-HER inhibitor dacomitinib. **a** Immunoblots of cell lysates evaluating ErbB signaling molecules from ALK1510-c4 cells treated with 1000 nM alectinib for 1, 3, 24, and 48 h and 9 days. **b** Relative HER2 phosphorylation levels in ALK1510-c4 cells to nontreated controls described in Fig. 3**a** were measured by ELISA (mean [SD] of *n* = 3 experiments). **c** Relative EGFR phosphorylation levels of ALK1510-c4 cells to total EGFR protein levels using cells described in Fig. 3**a** quantified by densitometric analysis with Sally Sue™. **d** Immunoblots of cell lysates from ALK1510-c4 DTP cells evaluating ErbB signaling and ALK signaling after treatment with 10, 100, and 1000 nM dacomitinib for 3 h. **e** Relative HER2 phosphorylation levels of ALK1510-c4 DTP cells described in Fig. 3**d** were measured by ELISA (mean [SD] of *n* = 3 experiments).

To evaluate whether ErbB signals contribute to the alectinib-induced DTP state, we performed time-dependence analysis of ErbB signals in ALK1510-c4 cells after treatment with 1000 nM alectinib. It showed transient activation of HER3 as phosphorylated HER3 increased from 24 to 48 h (Fig. 3a). Relative phosphorylation of HER2 was transiently increased by ~25% after exposure to 1000 nM alectinib for 24 h (Fig. 3b). EGFR remained in a phosphorylated state despite a decrease in total EGFR protein levels over 9 days of alectinib exposure. Thus, relative phosphorylation (phosphorylated EGFR [pEGFR]/total EGFR) increased approximately six-fold after 9 days of alectinib exposure, indicating that EGFR may be activated after 9 days of alectinib exposure (Fig. 3c). Further, ALK1510-c4 DTP cells treated with the pan-HER inhibitor dacomitinib (10, 100, and ‍1000 nM exposure) demonstrated a decrease in each phosphorylated level of EGFR, HER2, HER3, AKT and ERK and an increase in cleaved PARP levels (Fig. 3d, e). In line with the results of Fig. 3a, c, in RNA-seq analysis, EGFR mRNA expression levels were significantly decreased by treatment with alectinib at 24 h and 9 days, and the relative EGFR phosphorylation level was increased compared with nontreated controls (Supplementary Fig. 2a, b). These findings indicate that ALK1510-c4 DTP cells may activate ErbB signaling to evade alectinib-induced cell death.

### Efficacy and characterization of the combination of ALK-TKI with pan-HER inhibitors on ALK1510-c4 cells

**Fig. 4:**
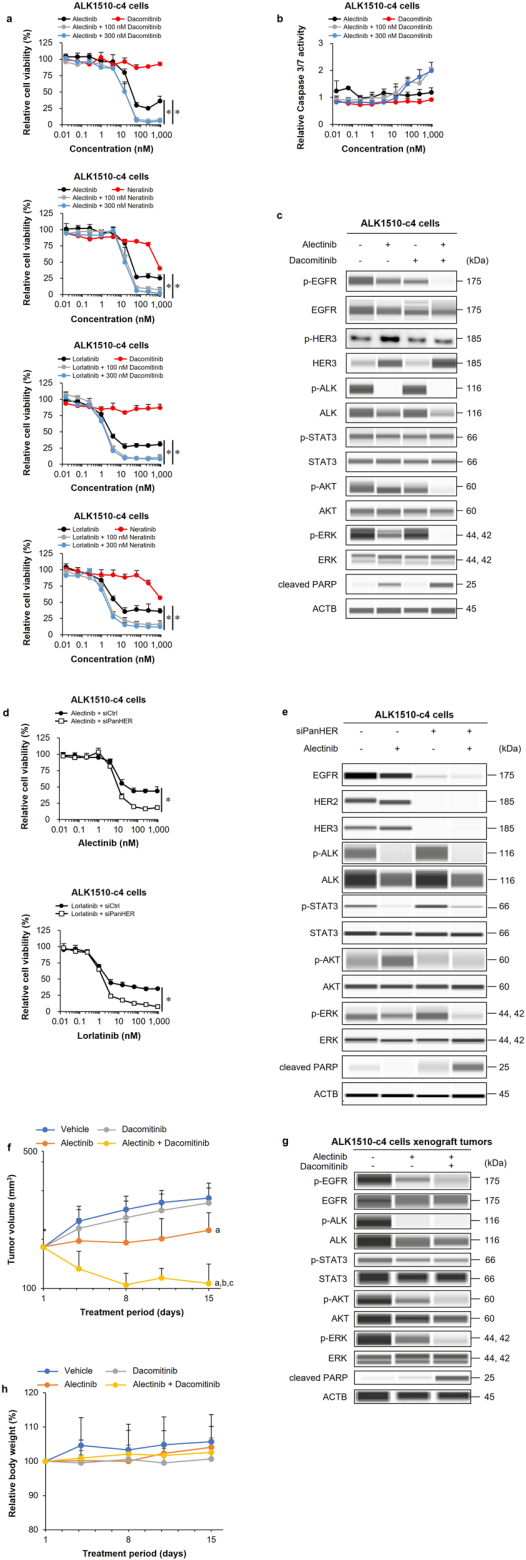
Efficacy and characterization of the combination of alectinib with pan-HER inhibitors on ALK1510-c4 cells. **a** Cell proliferation assay of ALK1510-c4 cells cultured with alectinib, lorlatinib, dacomitinib, and neratinib; and alectinib or lorlatinib in combination with dacomitinib or neratinib (100 nM and 300 nM) (mean [SD] of *n* = 3 experiments, *P < 0.05 between ALK-TKI single treatment and combination treatment; Dunnett’s test). **b** ALK1510-c4 cells cultured with alectinib, dacomitinib, or alectinib + dacomitinib (100 nM and 300 nM) were evaluated for caspase-3/7 activity relative to vehicle-treated cells (mean [SD] of *n* = 3 experiments). **c** Immunoblots of cell lysates evaluating ErbB and ALK downstream signaling in ALK1510-c4 cells treated with 1000 nM alectinib, 100 nM dacomitinib, and their combination for 24 h. **d** Cell proliferation assay of ALK1510-c4 cells transfected with three siRNAs against EGFR, HER2, and HER3, or a nontarget control siRNA and treated with alectinib or lorlatinib (mean [SD] of *n* = 3 experiments, *P < 0.05 between ALK-TKI + siCtrl and ALK-TKI + siPan-HER; Student’s *t* test). **e** Immunoblots of cell lysates from ALK1510-c4 cells described in Fig. 4d treated with 1000 nM alectinib for 48 h. **f** Mean (SD) change in tumor volume in mice bearing ALK1510-c4 cells xenograft tumors treated with vehicle, 2 mg/kg alectinib, 10 mg/kg dacomitinib, or their combination for 14 days (*P* < 0.05 versus vehicle (**a**), alectinib (**b**) and dacomitinib (**c**); Wilcoxon rank sum test by the Holm–Bonferroni method; *n* = 8 mice per group). **g** Immunoblots of tumor lysates from ALK1510-c4 cells xenograft tumors in mice treated with vehicle, alectinib (2 mg/kg), or alectinib (2 mg/kg) + dacomitinib (10 mg/kg) combination for 2 days. **h** Body weight change in mice relative to the start of the treatment in Fig. 4**f**.

Next, we examined whether the combination of a pan-HER inhibitor with ALK-TKIs prevents the development of ALK-TKI induced DTP and exerts a prominent combination effect. Cell proliferation assays showed that the combination treatment of alectinib or lorlatinib with two pan-HER inhibitors, dacomitinib or neratinib (at 100 nM and 300 nM concentrations), suppressed cell growth compared with alectinib or lorlatinib alone (Fig. 4a). The combination of alectinib with the pan-HER inhibitor dacomitinib (100 nM and 300 nM) increased caspase-3/7 activity, a marker of apoptosis, compared with alectinib alone, suggesting that the combination treatment induced apoptosis (Fig. 4b). Immunoblots of ALK1510-c4 cell lysates showed that the combination of alectinib with dacomitinib (100 nM) reduced phosphorylation of EGFR, HER3, AKT, and ERK, and increased cleaved PARP levels compared with alectinib alone (Fig. 4c). To confirm whether this combined efficacy is an effect of ErbB signal blockade, the sensitivity of ALK inhibitors was evaluated using siRNA that suppressed the expression of EGFR, HER2, and HER3. These siRNA treatments markedly reduced the protein levels of EGFR, HER2, and HER3 and increased the sensitivity to alectinib and lorlatinib. Compared with alectinib alone, these siRNA treatments decreased phosphorylated AKT and ERK levels, increased cleaved PARP levels, and induced apoptosis (Fig. 4d, e).

The subcutaneous ALK1510-c4 cell mouse xenograft model showed that the combination of alectinib (2 mg/kg) with dacomitinib (10 mg/kg) significantly suppressed tumor growth compared with single agents alone or vehicle (Fig. 4f). Immunoblots of tumor lysates from Fig. 4f show that the combination reduced the levels of phosphorylated EGFR, AKT, and ERK and increased the levels of cleaved PARP compared with alectinib alone (Fig. 4g). No weight loss was observed in mice treated with the combination of alectinib and dacomitinib or with either drug alone (Fig. 4h).

### Efficacy and characterization of the combination of ALK-TKI with pan-HER inhibitors on ALK0413 cells

To assess whether a similar DTP mechanism present in other ALK + NSCLC cells, we used ALK0413 cell lines established from another ALK+ NSCLC patient. Cell proliferation assays showed reduced sensitivity to alectinib in alectinib-induced ALK0413 DTP cells (ALK0413 DTP cells) established from ALK0413 cells after 13 days of 1000 nM alectinib exposure; culturing the cells for 35 days in the absence of alectinib restored alectinib sensitivity (Supplementary Fig. 3a).

Immunoblots show that alectinib exposure resulted in a transient increase in phosphorylated HER2 and HER3 levels at 1 and 3 h post-exposure, and after 13 days, phosphorylated EGFR levels increased. Phosphorylated ERK levels transiently decreased at 1 and 3 h and increased 13 days post-exposure. Cleaved PARP levels increased transiently at 3 h and gradually disappeared over 13 days of alectinib exposure (Supplementary Fig. 3b).

Cell proliferation assays showed that the combination of alectinib or lorlatinib with the pan-HER inhibitors dacomitinib or neratinib (100 nM and 300 nM) suppressed cell growth compared with alectinib or lorlatinib alone (Supplementary Fig. 3c). The combination of alectinib with the pan-HER inhibitor dacomitinib (100 nM and 300 nM) increased caspase-3/7 activity compared with that of alectinib or dacomitinib alone, suggesting that the combination treatment induced apoptosis (Supplementary Fig. 3d).

Immunoblots showed that the combination of 100 nM dacomitinib with alectinib reduced the levels of phosphorylated EGFR, HER2, HER3, AKT, and ERK and increased the levels of cleaved PARP compared with alectinib alone (Supplementary Fig. 3e). These findings are similar to those observed in ALK1510-c4 cells and indicate that a single-agent alectinib activates ErbB signals in a subpopulation of ALK0413 cells that can survive as DTP cells. Furthermore, the long-term assay showed that only the continuous combination reduced cell counts to bellow 1 × 10^4^ cells. In contrast, more than 1 × 10^5^ cells remained with alectinib therapy (Supplementary Fig. 3f, g). Thus, the combination of alectinib and a pan-HER inhibitor prevents ErbB signal activation to suppress the appearance of DTP cells, while inducing apoptosis to exert cytostatic effects.

### Characterization of TNKS1/2 pathway in ALK1510-c4 cells and ALK1510-c4 DTP cells

**Fig. 5:**
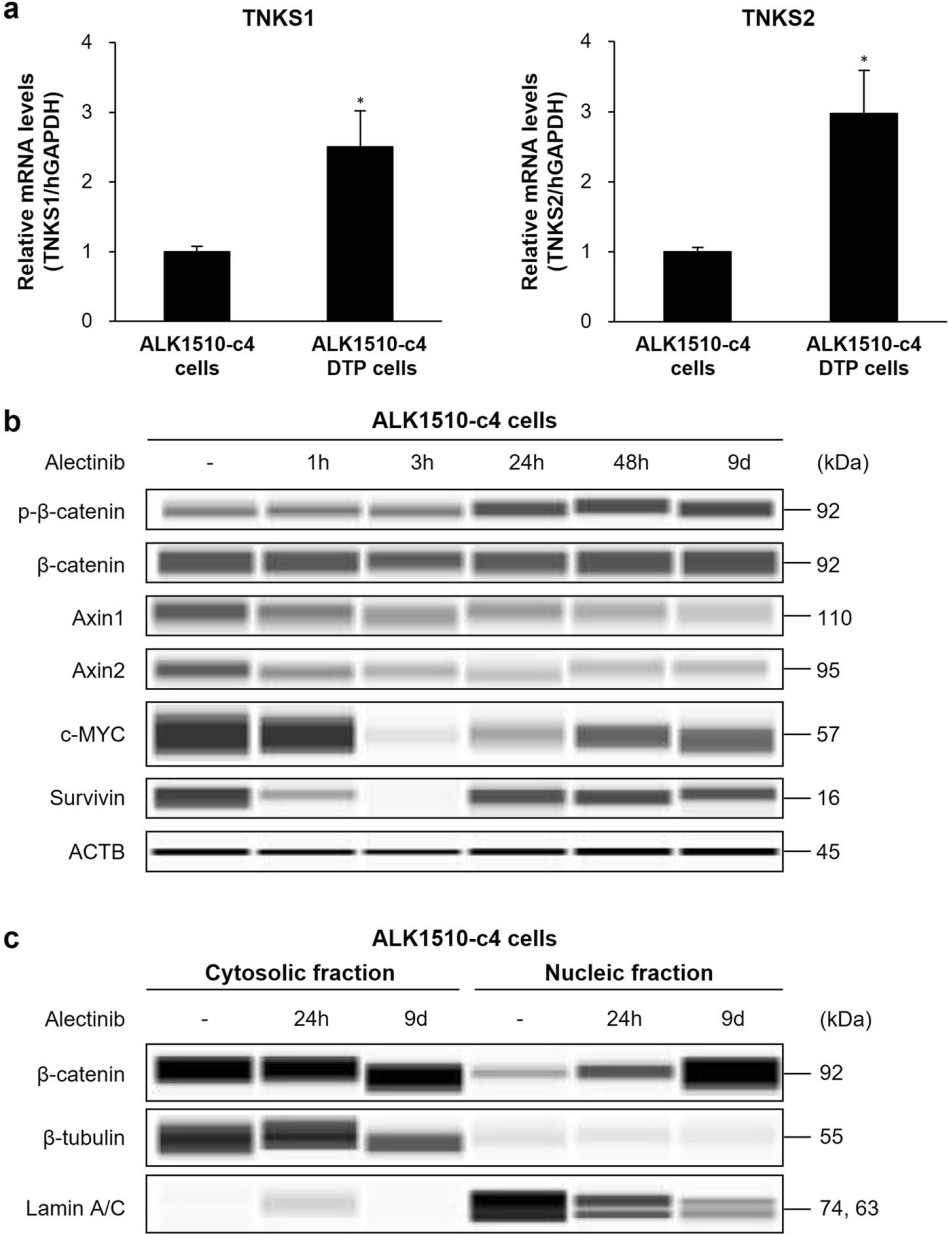
Characterization of TNKS1/2 pathway in ALK1510-c4 cells and ALK1510-c4 DTP cells. **a** The relative mRNA expression of TNKS1 and TNKS2 (TNKS1/2) in ALK1510-c4 cells and ALK1510-c4 DTP cells determined by RT-PCR (using the TaqMan probes) was calculated as the ratio of the normalized value (with GAPDH mRNA expression) (mean [SD] of *n* = 3 experiments; *P < 0.05 versus ALK1510-c4 cells; Dunnett’s test). **b** Immunoblots of cell lysates evaluating Wnt/β-catenin signaling in ALK1510-c4 cells treated with 1000 nM alectinib for 1, 3, 24, and 48 h and 9 days. **c** Immunoblots of cytosolic or nuclear lysates from ALK1510-c4 cells treated with 1000 nM alectinib for 24 h and 9 days.

Next, we analyzed the role of TNKS1/2, another candidate molecule identified in drug screening, in alectinib-induced DTP cells. TNKS1/2 are members of the PARP superfamily and one of their functions is the regulation of the canonical WNT/β-catenin signaling through modulation of β-catenin destruction^22^. ALK1510-c4 DTP cells have significantly elevated TNKS1 and TNKS2 mRNA levels compared with ALK1510-c4 cells (Fig. 5a). Immunoblots showed a decrease in Axin1/2 protein, one of the targets for degradation by TNKS1/2 and constituents of the β-catenin degradation complex, expression at all time points assessed (from 1 h to 9 days) and an increase in phosphorylated β-catenin expression (at Ser675, whereby β-catenin is activated and translocated to the nucleus) between 24 h and 9 days of alectinib exposure in ALK1510-c4 cells. Protein levels of survivin and c-MYC, whose transcription is controlled by various signaling pathways including β-catenin signaling, transiently decreased at 3 h after alectinib exposure and recovered after 24 h (Fig. 5b). Analysis of the subcellular localization of β-catenin showed that cytosolic β-catenin levels remained largely unchanged, whereas β-catenin levels in the nucleus were elevated with alectinib exposure, suggesting activation of nuclear translocation of β-catenin (Fig. 5c). Furthermore, N-Myc Downstream Regulated 1 (NDRG1) protein, which is upregulated by Wnt signaling^23^, was increased in ALK1510 DTP cells (Supplementary Fig. 4).

### Efficacy and characterization of TNKS1/2 inhibitors in ALK1510-c4 cells

**Fig. 6:**
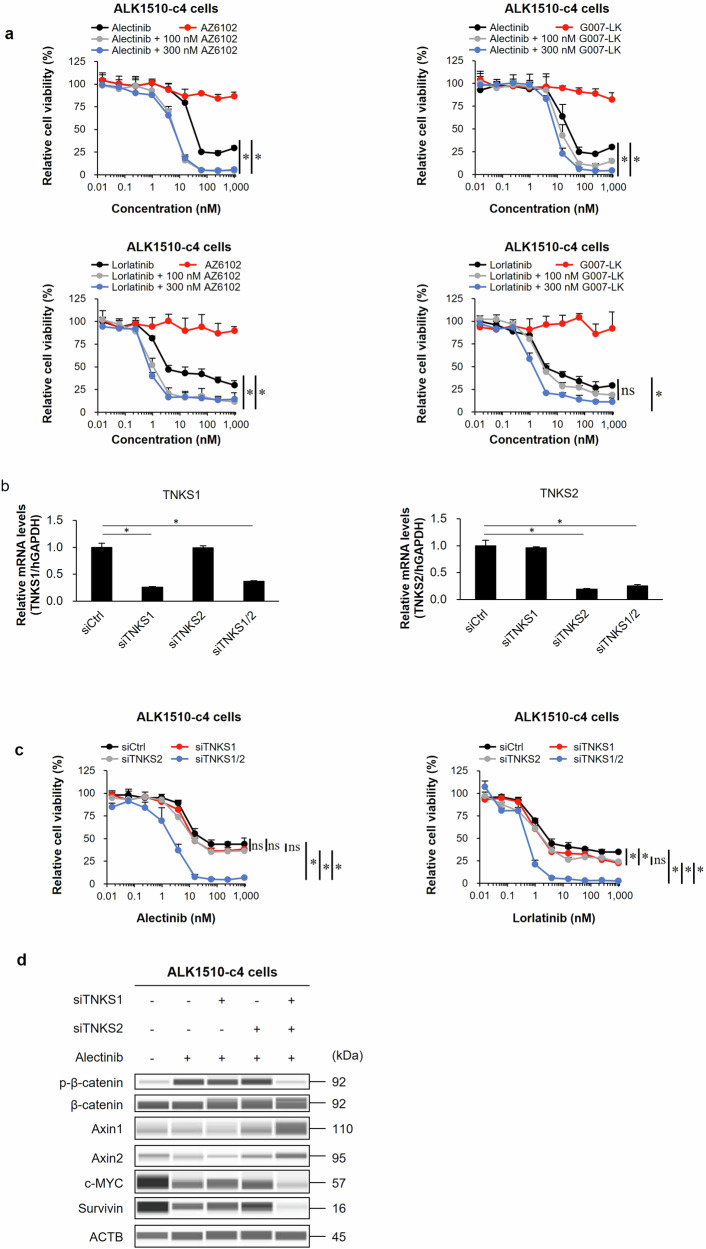
Efficacy in cell-based assay and characterization of TNKS1/2 inhibitors in ALK1510-c4 cells. **a** Cell proliferation assay of ALK1510-c4 cells cultured with alectinib, lorlatinib, AZ6102, or G007-LK singly, or alectinib or lorlatinib in combination with AZ6102 or G007-LK (100 nM or 300 nM) (mean [SD] of *n* = 3 experiments, *P < 0.05 between ALK-TKI single treatment and combination treatment; Dunnett’s test). **b** The relative mRNA expression of TNKS1/2 in ALK1510-c4 cells treated with 1000 nM alectinib was determined by RT-PCR (using the TaqMan probes) and calculated as the ratio of the normalized value with GAPDH mRNA expression (mean [SD] of *n* = 3 experiments, *P < 0.05 versus siCtrl; Dunnett’s test). **c** Cell proliferation assay of ALK1510-c4 cells transfected with two siRNAs against TNKS1, TNKS2, or a nontarget control siRNA and cultured with alectinib or lorlatinib (mean [SD] of *n* = 3 experiments, *P < 0.05 among siCtrl, siTNKS1, siTNKS2 and siTNKS1/2; Tukey’s HSD test). **d** Immunoblots assessing Wnt/β-catenin signaling molecules from cell lysates of ALK1510-c4 cells described in Fig. 6b treated with 1000 nM alectinib for 48 h.

To examine the efficacy of TNKS1/2 inhibitor, we performed a cell proliferation assay. The results showed that the combination of alectinib or lorlatinib with two TNKS1/2 inhibitors (AZ6102 and G007-LK [100 nM or 300 nM]) suppressed cell growth compared with alectinib or lorlatinib alone (Fig. 6a). To confirm whether the efficacy of this combination (ALK inhibitor with AZ6102) is due to TNKS1/2-mediated Wnt/β-catenin signaling blockade, we examined the sensitivity to ALK inhibitors using siRNAs that specifically suppressed the expression of TNKS1/2. The relative mRNA levels of TNKS1 and TNKS2 were significantly suppressed by each siRNA (Fig. 6b). The combination of the TNKS1 and TNKS2 siRNAs increased the sensitivity to alectinib and lorlatinib, while neither siTNKS1 nor siTNKS2 alone affected the growth inhibition effect of ALK-TKIs, suggesting that dual inhibition of TNKS1 and TNKS2 is necessary (Fig. 6c). Immunoblots showed that the combined use of these two TNKS1/2 siRNAs with alectinib decreased phosphorylated β-catenin levels, increased Axin1/2 protein levels, and decreased c-MYC and survivin protein levels compared with alectinib alone (Fig. 6d).

Further, we evaluated the effect of β-catenin knockdown on DTP formation. As we anticipated, the expression level of c-MYC was downregulated in siCTNNB1-treated cells (Supplementary Fig. 5a). Also, following treatment with 1000 nM alectinib for 9 days, cell growth was significantly inhibited in siCTNNB1-treated cells compared to siCtrl-treated cells (Supplementary Fig. 5b).

**Fig. 7:**
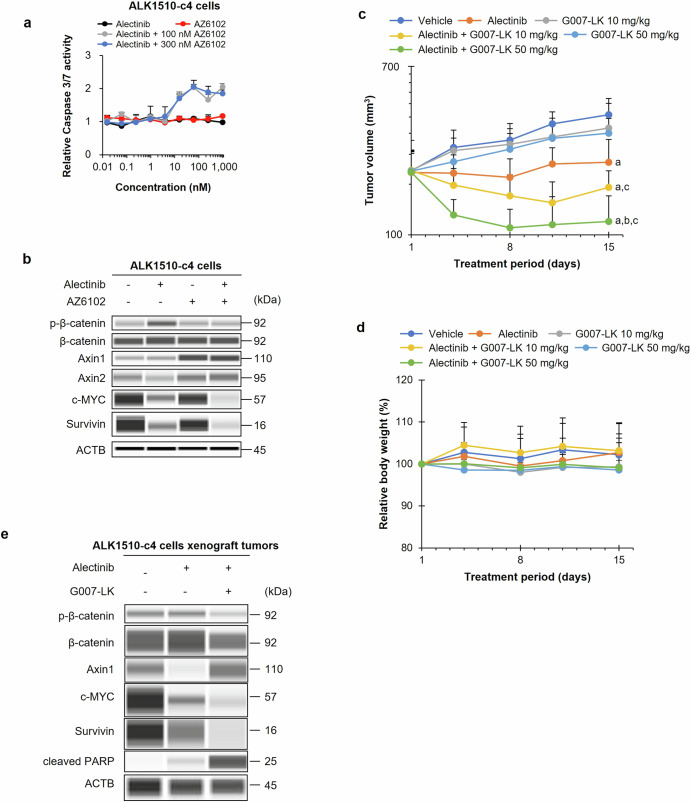
Efficacy in animal model and characterization of TNKS1/2 inhibitors in ALK1510-c4 cells. **a** Assessment of caspase-3/7 activity in ALK1510-c4 cells cultured with alectinib, AZ6102, and the combination of alectinib and AZ6102 (100 nM or 300 nM) relative to caspase-3/7 activity in vehicle-treated cells (mean [SD] of *n* = 3 experiments). **b** Immunoblots assessing Wnt/β-catenin signaling molecules from cell lysates of ALK1510-c4 cells treated with alectinib (1000 nM), AZ6102 (100 nM), or their combination for 48 h. **c** Mean (SD) change in tumor volume in mice bearing xenograft tumors with ALK1510-c4 cells and treated with vehicle, 2 mg/kg alectinib, 10 mg/kg or 50 mg/kg of G007-LK, or their combinations for 14 days (*P* < 0.05 versus vehicle (**a**), alectinib (**b**) and corresponding dose of G007-LK (**c**); Wilcoxon rank sum test by the Holm–Bonferroni method; *n* = 6 mice per group). **d** Change in body weight from baseline of mice treated with vehicle, alectinib (2 mg/kg), G007-LK (10 mg/kg and 50 mg/kg), and a combination of alectinib (2 mg/kg) and G007-LK (10 mg/kg or 50 mg/kg). **e** Immunoblots of tumor lysates from mice bearing xenograft tumors with ALK1510-c4 cells and treated with alectinib (2 mg/kg), or a combination of alectinib (2 mg/kg) and G007-LK (10 mg/kg) for 2 days.

The combination of alectinib with AZ6102 (100 nM or 300 nM) increased caspase-3/7 activity compared with that of alectinib or AZ6102 alone, suggesting that the combination treatment induced apoptosis (Fig. 7a). The combination of alectinib with AZ6102 increased the protein expression of Axin1/2, suppressed phosphorylated β-catenin levels, and suppressed protein levels of c-MYC and survivin compared with alectinib alone (Fig. 7b).

The tumor volume of mice with ALK1510-c4 cells xenograft tumors was significantly reduced with the combination treatment of alectinib and G007-LK (10 and 50 mg/kg) compared with the treatment with alectinib alone without weight loss (Fig. 7c, d). The combination treatment of G007-LK with alectinib increased Axin1 protein levels, decreased phosphorylated β-catenin levels that were increased by alectinib alone, and decreased c-MYC and survivin protein levels compared with alectinib alone (Fig. 7e).

### Efficacy and characterization of triple combination therapy in ALK1510-c4 cells and animal models

**Fig. 8:**
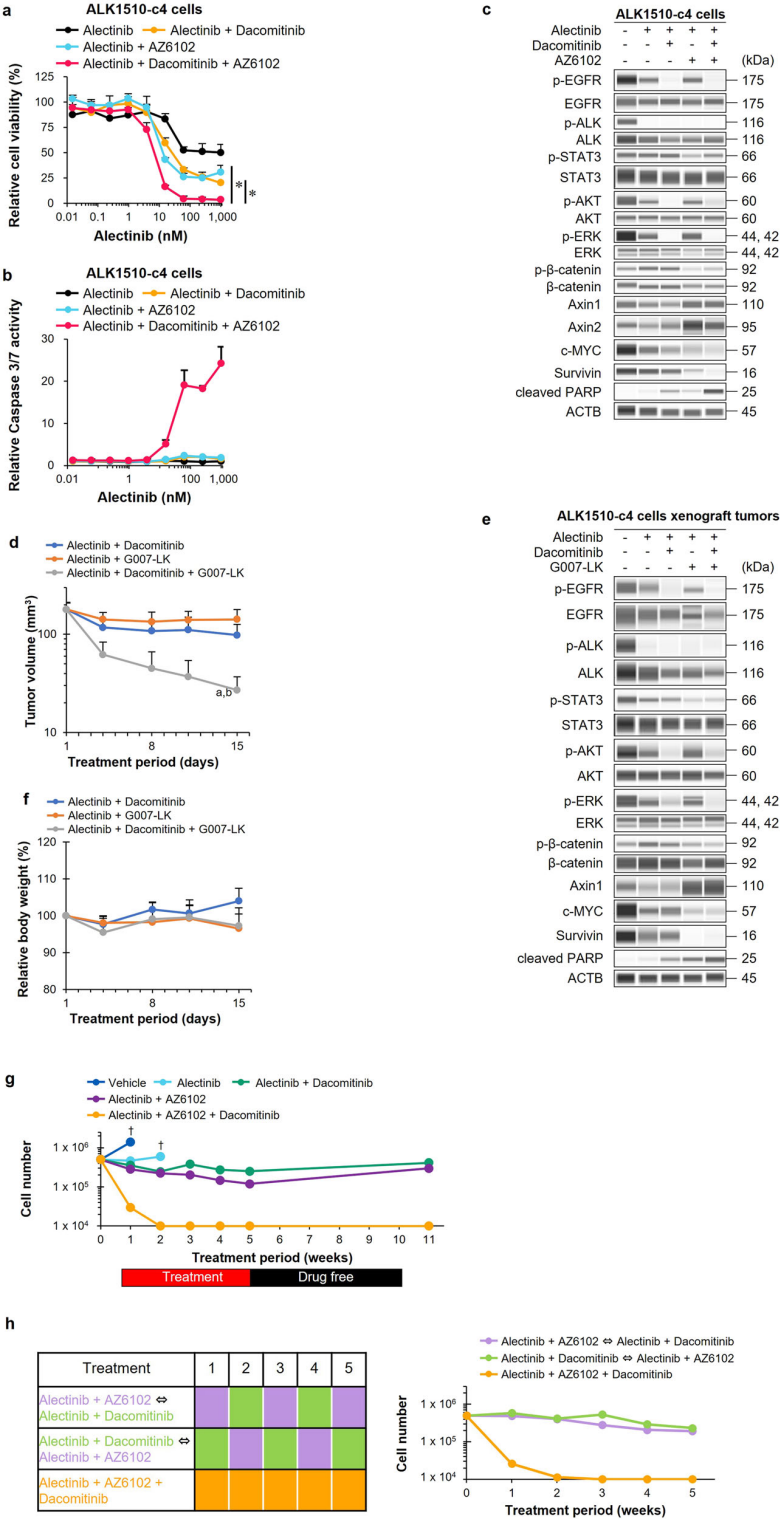
Efficacy and characterization of triple combination therapy in ALK1510-c4 cells and animal models. **a** Relative cell viability of ALK1510-c4 cells cultured with alectinib, alectinib with dacomitinib (100 nM), alectinib with AZ6102 (100 nM), and a triple combination of alectinib + dacomitinib + AZ6102 (mean [SD] of *n* = 3 experiments, *P < 0.05 between doublet treatment and alectinib + dacomitinib + AZ6102 treatment; Dunnett’s test). **b** Caspase-3/7 activity relative to vehicle treatment was assessed in ALK1510-c4 cells treatment with the drugs described in Fig. 8**a** (mean [SD] of *n* = 3 experiments). **c** Immunoblots of cell lysates from ALK1510-c4 cells described in Fig. 8**a** after treatment for 24 h. **d** Mean (SD) change in tumor volume of mice bearing xenograft tumors with ALK1510-c4 cells and treated with vehicle, alectinib (2 mg/kg) + dacomitinib (10 mg/kg), alectinib (2 mg/kg) + G007-LK (10 mg/kg), and their triple combination for 14 days (*P* < 0.05 versus alectinib + dacomitinib (**a**) or versus alectinib + G007-LK (**b**) ; Wilcoxon rank sum test by the Holm–Bonferroni method; *n* = 8 mice per group). **e** Immunoblots of tumor lysates evaluating ALK-signaling and Wnt/β-catenin-signaling from xenograft tumors with ALK1510-c4 cells in mice treated with alectinib, alectinib + dacomitinib, alectinib + G007-LK, and their triple combination for 2 days. **f** Mean (SD) change from baseline in body weight of mice described in Fig. 8d. **g** ALK1510-c4 cells were treated with vehicle, alectinib (1000 nM), alectinib in combination with dacomitinib (100 nM) or AZ6102 (100 nM); and a triple combination of alectinib + dacomitinib + AZ6102 for 5 weeks, and then washed after 5 weeks. Cell numbers were measured using a cell counter at weeks 1, 2, 3, 4, 5, and 11. If the cell number was 1 × 10^4^ or less, it was indicated and considered a limitation of the cell counter. The cell counts were terminated at the point (†) when ALK1510-c4 cells detached from the culture flask due to over-confluence (*n* = 1). **h** ALK1510-c4 cells were sequentially treated with alectinib (1000 nM) in combination with dacomitinib (100 nM) or AZ6102 (100 nM), and a triple combination of alectinib + dacomitinib + AZ6102 for 5 weeks (left). Cell numbers were measured using a cell counter at weeks 1, 2, 3, 4, and 5 (right). If the cell number was 1 × 10^4^ or less, it was indicated and considered a limitation of the cell counter (*n* = 1).

The relative cell viability of ALK1510-c4 cells treated with the triple combination of alectinib, dacomitinib, and AZ6102 showed that, compared with the dual combinations of alectinib and dacomitinib or alectinib and AZ6102, the triple combination inhibited cell growth (Fig. 8a). Dual combination therapy with AZ6102 and dacomitinib did not inhibit cell growth, in contrast to triple drug therapy in ALK1510-c4 cells (Supplementary Fig. 6a). We additionally confirmed that triple combination did not induce further growth suppression in A549 cells, which are ALK negative KRAS G12S positive NSCLC cells, compared with alectinib single treatment. (Supplementary Fig. 6b). The triple combination also increased caspase-3/7 activity, suggesting a strong induction of apoptosis compared with the dual combinations (Fig. 8b and Supplementary Fig. 6c). Immunoblots showed that the triple combination therapy decreased the levels of phosphorylated EGFR, AKT, and ERK similar to those observed with the dual combination of alectinib and dacomitinib. The triple combination also increased Axin1/2 levels to almost the same level as observed with the dual combination of alectinib and AZ6102 and lowered the phosphorylated β-catenin, survivin, and c-MYC protein levels compared with the dual combination of alectinib and AZ6102. The triple combination resulted in higher cleaved PARP levels, suggesting stronger induction of apoptosis than the dual combinations (Fig. 8c).

The triple combination significantly suppressed ALK1510-c4 cell xenograft tumor growth in mice compared with the dual combinations of alectinib and dacomitinib or alectinib and G007-LK (Fig. 8d). Immunoblots showed that the triple combination decreased the levels of phosphorylated EGFR, AKT, and ERK to the same level as that observed with the dual combination with alectinib and dacomitinib. The triple combination also increased Axin1 levels to almost the same level as observed with the dual combination of alectinib and AZ6102, and decreased phosphorylated β-catenin, survivin, and c-MYC protein levels in comparison with the dual combination of alectinib and AZ6102. The triple combination also resulted in increased cleaved PARP levels, suggesting a stronger induction of apoptosis than the dual combinations (Fig. 8e). No reduction in body weight was observed with the triple combination compared with the dual combinations in mice (Fig. 8f).

We examined if the efficacy of the triple combination was sustained with an extended exposure for 5 weeks in in vitro setting. Although the number of cells in the vehicle or alectinib alone groups increased beyond the detection limit at 1 or 2 weeks after the start of culture and 1 × 10^5^ or more cells remained in both dual-drug combinations after 5 weeks of exposure, in the triple-drug combination, the cell counts fell to less than 1 × 10^4^ cells after 2 weeks of exposure and remained below the detectable limit. Compared with the dual-drug combinations, the triple-drug combinations strongly suppressed cell growth even over 5 weeks of exposure. With the triple combination, no regrowth of cells was observed when cultured in a drug-free medium after 5 weeks, indicating that the 5-week triple combination treatment may have led to complete cell death (Fig. 8g). Furthermore, we assessed whether the sequential treatment of doublet therapy was comparable to triple combination. As shown in Fig. 8h, the cell counts of only the triple combination fell to less than 1 × 10^4^ cells. In contrast, more than 1 × 10^5^ cells remained with both sequential doublet therapies.

## Discussion

This study explored novel dual or triple combination therapies that have the potential to inhibit the formation of ALK1510-c4 DTP cells from ALK1510-c4 cells obtained from patient with ALK+ NSCLC. Drug-library screening and reproducible experiments suggested that pan-HER inhibitors and TNKS1/2 inhibitors were likely to inhibit the formation of ALK1510-c4 DTP cells by inhibiting cell growth signals activated by ErbB or Wnt/β-catenin signaling pathways, respectively.

The hallmarks of DTP cells, such as the restoration of sensitivity to alectinib in an alectinib-free medium; increased expression of BIM, CD133, and vimentin; and decreased expression of E-cadherin, were confirmed^24–26^. Our findings suggest that in the early stages of alectinib exposure, ALK signaling blockade induces cell death in ALK1510-c4 cells; ALK1510-c4 DTP cells showed reactivation of the downstream ALK signaling molecules (AKT and ERK). ALK1510-c4 DTP cells also showed significantly elevated TNKS1 and TNKS2 mRNA levels compared with ALK1510-c4 cells.

ErbB signals contributing to the DTP state were a transient increase in phosphorylated HER3 and HER2 and persistence of phosphorylated EGFR, indicating that EGFR may be activated after prolonged alectinib exposure. The pan-HER inhibitor dacomitinib was shown to decrease pEGFR, pHER2, and pHER3 levels in the ErbB signaling pathway along with a decrease in pAKT and pERK levels and an increase in cleaved PARP levels in the ALK signaling pathway in ALK1510-c4 DTP cells. This suggested that DTP cells may activate ErbB signaling to evade alectinib-induced cell death. The combination of alectinib and a pan-HER inhibitor prevented the development of DTP cells by blocking ErbB signal activation and induced apoptosis to exert the antitumor effect.

Subsequently, the efficacy of the dual combination treatment of alectinib or lorlatinib with two pan-HER inhibitors dacomitinib or neratinib was confirmed by a suppression of cell growth using two ALK + NSCLC lung cancer cell lines and reduction in subcutaneous ALK1510-c4 cell xenograft tumors in mice. Such a combination effect was also confirmed using siRNAs targeting the ErbBs family proteins. As with our results, Tanimura et al. have demonstrated that the combination therapy of ALK-TKIs with the pan-HER inhibitor afatinib increased cell sensitivity to the ALK-TKIs in ALK + NSCLC cell lines with a mesenchymal-like phenotype and prevented tumor regrowth in mouse xenograft models through the prevention of the development of ALK-TKI-tolerant cells by HER3 activation^14^. Also, Katayama et al. have reported that the ALK-TKI resistance mechanism through EGFR and its ligands^27^. Among the EGFR and HER3 ligands, mRNA expression of EGF and neuregulin 2 (NRG2) were notably elevated, exhibiting a more than two-fold increase in DTP cells as compared to in ALK1510-c4 cells (Supplementary Fig. 7a). However, the level of EGF protein was not increased in DTP cells, and NRG2 protein was not detected in western blotting (Supplementary Fig. 7b), suggesting that ErbB signals may be activated by mechanisms other than those associated with of EGFR and HER3 ligands.

TNKS1/2 is one of the proteins that constitutes Wnt/β-catenin signaling, and TNKS1/2 binds Axin1/2 to promote Axin1/2 degradation. β-catenin has a function as a transcription factor working with TCF/LEF that controls the transcription of various antiapoptotic and cell growth-related genes, but it is degraded and inactivated via the ubiquitin-proteasome pathway by Axin1/2. Wnt signaling activation promotes the degradation of Axin1/2 by TNKS1/2 and suppresses the degradation of β-catenin, thereby activating the nuclear translocation of β-catenin and the transcription of various antiapoptotic and proliferation-related genes (*BIRK5,* also called *survivin,* and *c-MYC*)^28–30^. Wnt/β-catenin signals identified in ALK1510-c4 cells that helped DTP cells evade cell death induced by alectinib could be attributed to increased expression levels of TNKS1/2 and the accelerated degradation of Axin1/2 9 days after alectinib exposure in ALK1510-c4 cells. Consequently, the degradation of β-catenin is suppressed, resulting in an increase in the nuclear translocation of β-catenin, which in turn activates the transcription of survivin and c-MYC, leading to an increase in the levels of these proteins. Accordingly, the efficacy of the dual combination treatment of alectinib or lorlatinib with two TNKS1/2 inhibitors (AZ6102 and G007-LK) was confirmed by strong suppression of cell growth and reduction in subcutaneous ALK1510-c4 cell xenograft tumors in mice. The mechanism of action was confirmed using siRNAs that specifically suppressed the expression of TNKS1/2 and increased the sensitivity to the ALK inhibitors alectinib and lorlatinib. These findings indicate that ALK1510-c4 cells survive as DTP cells by activating Wnt/β catenin signaling via TNKS1/2 with alectinib alone, but in combination with TNKS1/2 inhibitors, the Wnt/β-catenin signaling activation is inhibited to suppress the appearance of DTP cells, thereby strongly inducing apoptosis to exert a cytostatic effect.

The TNKS1 and TNKS2 are members of the PARP superfamily of enzymes. While the molecular mechanisms of TNKS1 have be studied more extensively than those of TNKS2, some level of functional redundancy is suggested between the two enzymes^31^, and TNKS1 and TNKS2 have high homology^32^. Tankyrase inhibitors inhibit both TNKS1 and TNKS2 and contribute to the suppression of the Wnt/β-catenin signaling^33^. Furthermore, similar to our results, other studies have also reported that the knockdown of TNKS1 or TNKS2 alone using siRNA is insufficient to increase Axin levels and attenuate the Wnt/β-catenin signaling and that simultaneous knockdown of both TNSK1 and TNSK2 is required^22^. These results suggest the importance of combination with alectinib with compounds that inhibit both TNKS1 and TNKS2. The remaining cells after 9 days of treatment with alectinib + dacomitinib showed a significant reduction in EGFR activation but no reduction in c-Myc protein levels compared to alectinib alone (Supplementary Fig. 8a). On the other hand, 9 days of treatment with alectinib + AZ6102 showed no significant reduction in EGFR activation and no reduction in c-Myc protein levels compared to alectinib alone (Supplementary Fig. 8b). These results suggest that HER-driven adaptation and WNT-driven adaptation likely contribute to DTP emergence independently, rather than through a direct interaction between the two pathways. The results of the triple combination therapy compared with each dual combination therapy suggest that the ErbB pathway and the Wnt/β-catenin pathway (or at least their downstream signals) are activated independently of each other. The triple combination therapy showed greater inhibition of cell growth, increase in caspase-3/7 activity, and suppression of ALK1510-c4 cell xenograft tumor growth compared with dual combination therapy. The antitumor effects of inhibiting two pathways with the triple combination therapy were stronger and sustained after an extended exposure compared with the dual combinations. To the best of our knowledge, this is the first report to show that there are multiple different DTP mechanisms in a single cell type and that simultaneous inhibition of each mechanism leads to superior combined effects compared with single or double inhibition.

TNKS1/2 inhibitors have not been used clinically because of their gastrointestinal toxicity. However, it is also known that abnormal β-catenin signaling is associated with poor prognosis in NSCLC in clinical practice^34^. In recent years, TNKS1/2 inhibitors that suppress on-target gastrointestinal toxicity have been developed till the nonclinical level^35^. There are several reports showing excellent antitumor effects when combined with EGFR inhibitors and PI3K inhibitors^36,37^. Furthermore, our study showed that the combination of TNKS1/2 inhibitors and ALK-TKIs exerted excellent antitumor effects. This highlights the importance of the development of TNKS1/2 inhibitors that can be used in clinical practice.

XAV939, a small-molecule TNKS1 inhibitor, was shown to promote β-catenin degradation^22^, which is similar to the suppression of phosphorylated β-catenin levels observed with the alectinib + TNKS1/2 inhibitor combination in our study. The suppression of cell growth with alectinib + TNKS1/2 inhibitor was similar to those observed with XAV939 in neuroblastoma cell lines^38^, lung adenocarcinoma cells^39^, and small cell lung cancer cells in vitro^40^. The combination of TNKS1/2 inhibitor + EGFR inhibitor was also shown to induce antiproliferative activity in NSCLC cells^41^.

In ALK + NSCLC, acquired resistance is an unmet need, and early and effective combination therapy in a first-line setting might prevent the occurrence of DTP state and treat the acquired resistance. Combination therapies of TKI with mitogen-activated protein kinase (MEK) inhibitors, angiogenesis inhibitors, and chemotherapy are being evaluated in Phase 1/1b/2 trials mostly in the second-line and beyond treatment settings for the treatment of ALK + NSCLC; a few trials are also evaluating combination therapy in treatment-naive patients^42^. Similarly, pan-HER inhibitors and TNKS1/2 inhibitors are being evaluated in clinical trials as monotherapy or in combination with other drugs, such as MET inhibitors, in patients with advanced cancers or disease progression after >2 previous treatment regimens^43–46^.

There are five limitations in this study: 1) The detailed mechanism for how and whether Wnt pathway was activated in DTP cells remains unclear. However, some studies have suggested that dysregulation of the Wnt pathway may be an important factor contributing to enhanced maintenance and proliferation signaling in various cancers^47,48^, and crosstalk between EGFR and the Wnt signal pathway may enhance lung cancer tumorigenesis^47,49,50^. Furthermore, relative EGFR phosphorylation level to total EGFR expression level was upregulated in ALK1510-c4 DTP cells. Chaudhary et al. have reported that while EGFR protein level was downregulated due to NDRG1 mediated degradation, the activation of EGFR and its downstream signals were maintained via EGFR-Scr signaling in Triple negative breast cancer DTP cells^51^. Our study also showed increased expression of NDRG1, suggesting that crosstalk between EGFR and Wnt pathways may occur in ALK1510-c4 DTP cells. 2) The criteria for target protein expression levels that may predict the efficacy of dual and triple combination therapies remain unclear. It is unknown whether all patients with ALK lung cancer should be treated with dual or triple therapy regimens, or whether this treatment paradigm should be reserved for a more select group of patients with ALK fusion–positive lung cancer. 3) Weight loss was not observed in the mouse model, but it is not sufficient to assess the potential side effects associated with the dual and triple therapy regimens by evaluating the mouse model weight. A clinical trial of combination therapy with crizotinib and erlotinib revealed several adverse events^52^. Furthermore, diarrhea and skin related adverse events have occurred in dacomitinib-treated patients^53^. Although such symptons were not observed our in vivo studies during general observation in combination treatments, to more fully evaluate the safety of the combination therapy, additional in vitro and in vivo models, such as an in vitro ileal model^54,55^, epidermis model^56^ and a monkey model, will be required. 4) Including ALK + NSCLC patients, it is quite difficult to obtain DTP cells from patient samples. In the future, we need to evaluate whether the ErbB pathway and Wnt pathway develop DTP cells using specimen taken from patients with DTP-like SD status which would mean that tumor size reduction has ceased. 5) Our phosphoproteomic analysis could not anticipate the activation of Wnt and EGFR signaling. For deep phosphoproteomics analysis, it may be necessary to perform immunoprecipitation to concentrate the target protein or increase the sample volume. Also, DIA mass spectrometry may be useful for robust profiling^57^. On the other hand, our phosphoproteomics analysis suggested that other mechanisms may be involved in DTP and drug resistance. For example, phosphorylated PAK1 was significantly upregulated in DTP cells compared to cells treated with alectinib for 3 h (Supplementary Fig. 1c), and previous research has suggested that the CD44/PAK1/AKT axis induces drug resistance in squamous-cell lung cancer^58^. We plan to assess whether these factors confer ALK-TKI induced DTP status and resistance in a future study.

The results of this study indicate that ALK1510-c4 cells survive as DTP cells by activating ErbB signaling and Wnt/β-catenin signaling via TNKS1/2 when exposed to alectinib alone. The combination of alectinib and pan-HER inhibitors inhibits the appearance of DTP cells by blocking ErbB signaling activation and induces apoptosis to exert a cytostatic effect. Also, in combination with TNKS1/2 inhibitors, the Wnt/β-catenin signaling activation is inhibited to suppress the appearance of DTP cells, again inducing apoptosis to exert a cytostatic effect. Moreover, the triple combination therapy of alectinib + pan-HER inhibitor + TNKS1/2 inhibitor has the potential to inhibit cell proliferation and induce apoptosis more strongly than the dual combination even after an extended exposure. Our study suggested that targeting both pathways can be applied as a first-line treatment strategy for prevention of the DTP cell state and may lead to overcoming further treatment resistance.

## Supplementary information

supplementary file

## Data Availability

For further details on Chugai’s Data Sharing Policy and how to request access to related clinical study documents, see here (www.chugai-pharm.co.jp/english/profile/rd/ctds_request.html). All experimental data supporting the findings of this study are available within the article and its supplementary information files. The phosphoproteomics data used in this manuscript have been deposited to the jPOSTrepo with the identifier JPST003007.
