# Supplementary material for: Targeting ErbB and tankyrase1/2 prevent the emergence of drug-tolerant persister cells in ALK-positive lung cancer
Source: NPJ Precis Oncol. 2024 Nov 17;8:264. doi: 10.1038/s41698-024-00757-w (PMC11570601; doi:10.1038/s41698-024-00757-w)

Supplementary Figure 1

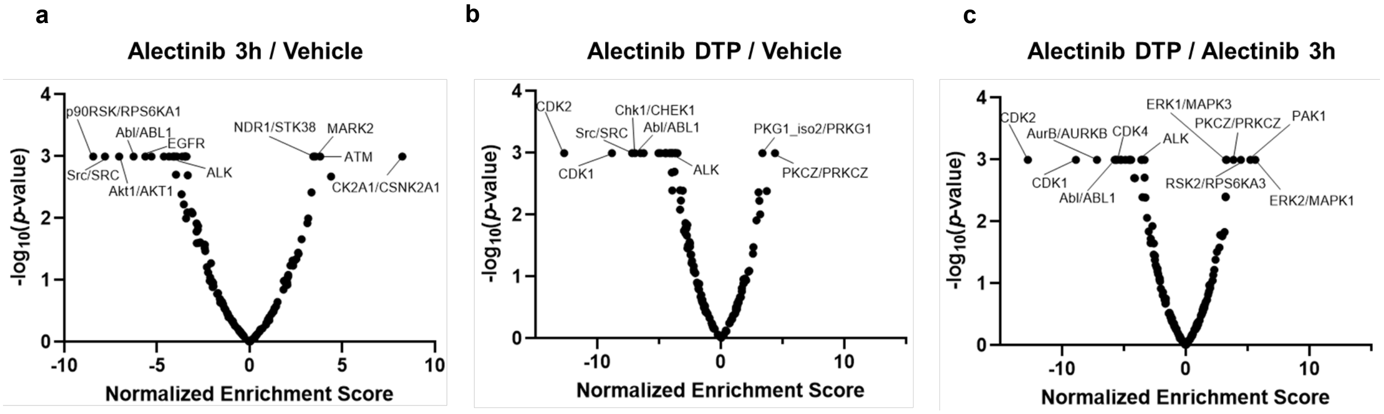

Supplementary Figure 1. Phosphoproteomics analysis indicated suppression of pALK in alectinib treated cells. (a-c) Volcano plot displaying the $-\log_{10}$(*p*-value) versus normalized enrichment score for (a) Alectinib 3h / Vehicle, (b) Alectinib DTP / Vehicle and (c) Alectinib DTP / Alectinib 3h.

Supplementary Figure 2

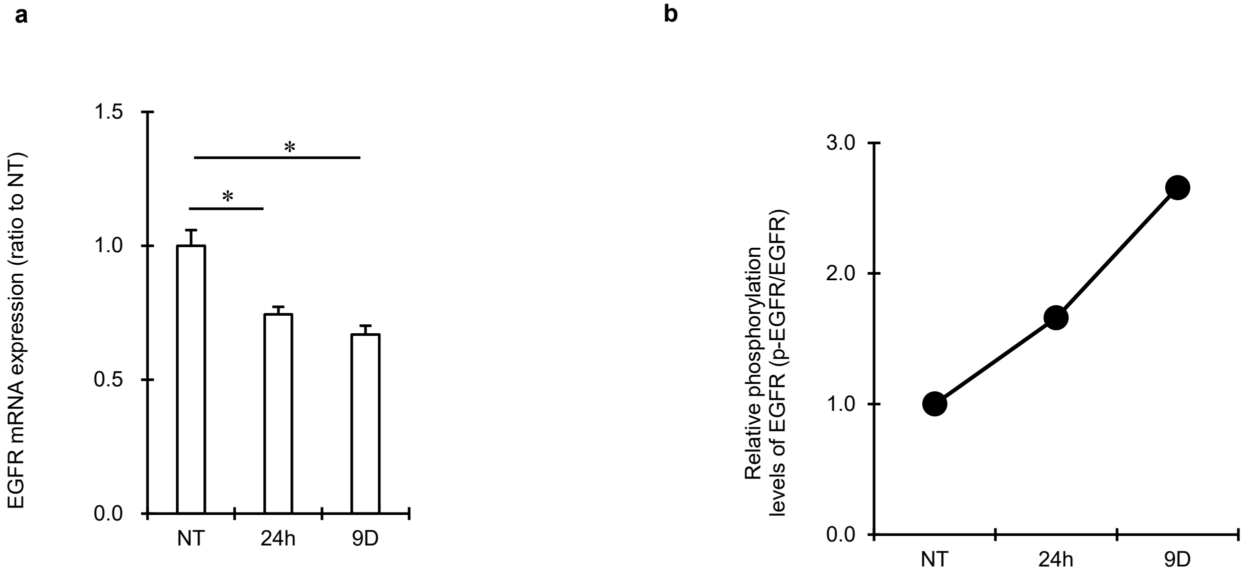

Supplementary Figure 2. EGFR phosphorylation level was upregulated after 9 days of alectinib exposure.

(a) Relative EGFR mRNA expression levels in ALK1510-c4 cells treated with 1,000 nM alectinib for 24 h and 9 days to nontreated controls were measured by RNA sequence (mean [SD] of n = 3 experiments). *$P < 0.05$ between alectinib at 24 h or 9 days versus nontreated controls in the Student's *t*-test by the Holm method. (b) Relative EGFR phosphorylation levels to EGFR mRNA expression levels of ALK1510-c4 cells described in Fig. S2a.

Supplementary Figure 3

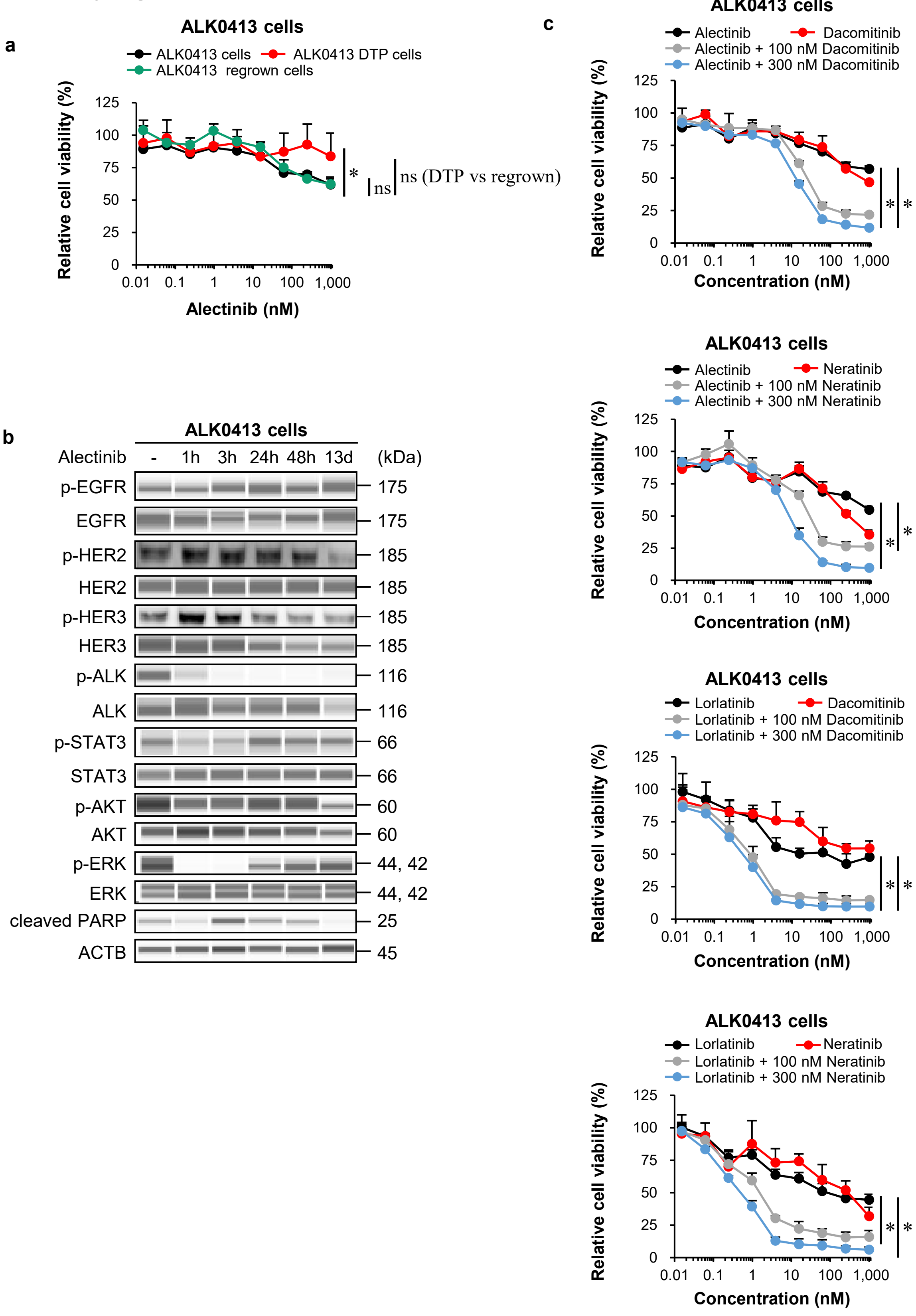

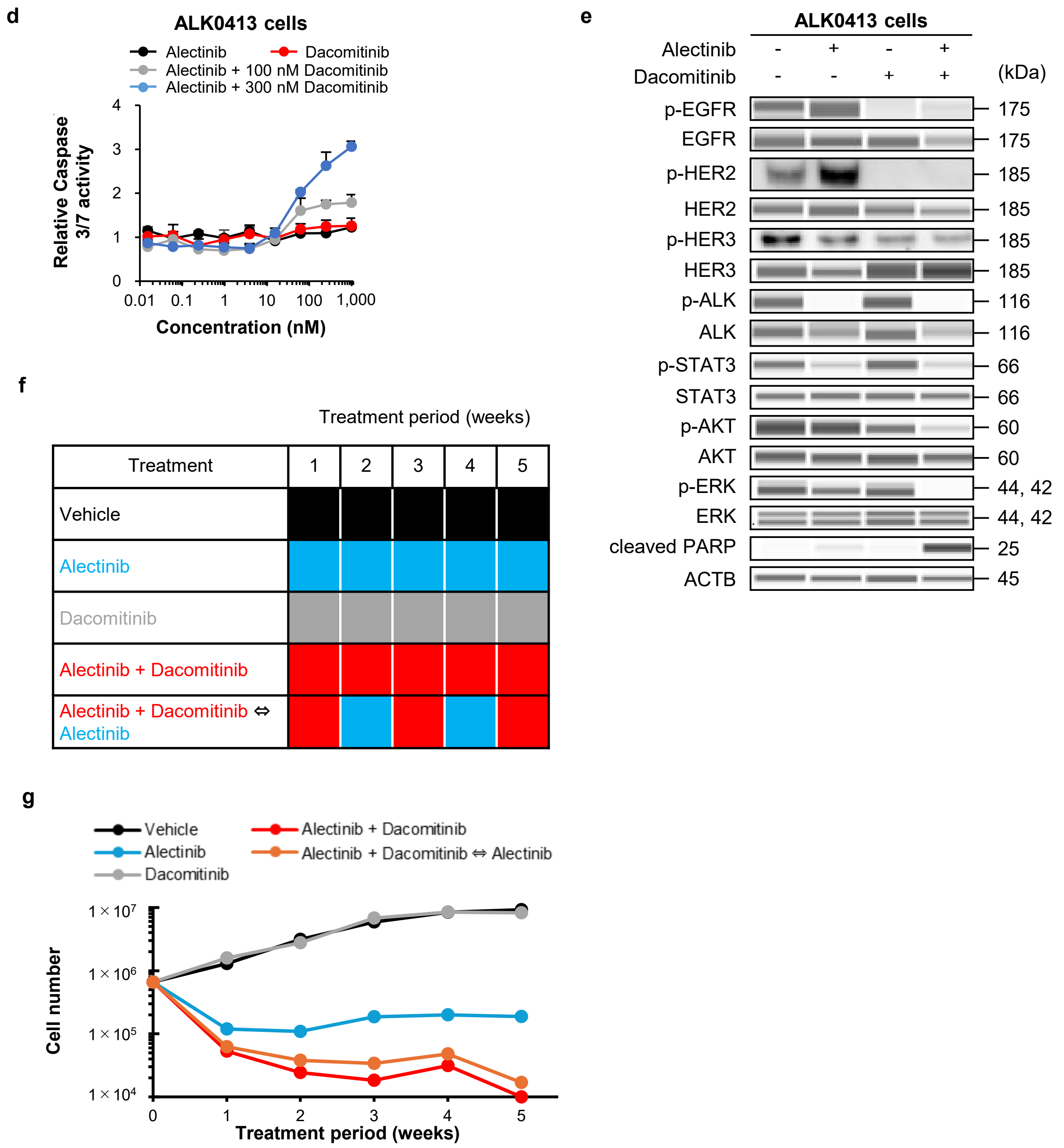
d
ALK0413 cells
Alectinib
Dacomitinib
Alectinib + 100 nM Dacomitinib
Alectinib + 300 nM Dacomitinib
Relative Caspase 3/7 activity
0
1
2
3
4
0.01
0.1
1
10
100
1,000
Concentration (nM)
e
ALK0413 cells
Alectinib - + - +
Dacomitinib - - + +
(kDa)
p-EGFR 175
EGFR 175
p-HER2 185
HER2 185
p-HER3 185
HER3 185
p-ALK 116
ALK 116
p-STAT3 66
STAT3 66
p-AKT 60
AKT 60
p-ERK 44, 42
ERK 44, 42
cleaved PARP 25
ACTB 45
f
Treatment period (weeks)
Treatment
1
2
3
4
5
Vehicle
Alectinib
Dacomitinib
Alectinib + Dacomitinib
Alectinib + Dacomitinib ⇔ Alectinib
g
Vehicle
Alectinib
Dacomitinib
Alectinib + Dacomitinib
Alectinib + Dacomitinib ⇔ Alectinib
Cell number
1 × 10^7
1 × 10^6
1 × 10^5
1 × 10^4
0
1
2
3
4
5
Treatment period (weeks)

Supplementary Figure 3. Efficacy and characterization of pan-HER inhibitors on ALK0413 cells.

(a) Alectinib-induced ALK0413 DTP cells (ALK0413 DTP cells) from a different ALK+ lung cancer cell line (ALK0413 cells) after treatment with 1000 nM alectinib for 13 days, ALK0413 regrown cells from ALK0413 DTP cells cultured in alectinib-free cell culture for 35 days and its comparison with ALK0413 cell controls were assessed in cell proliferation assay (mean [SD] of n = 3 experiments, $*P < 0.05$ between ALK-TKI single treatment and combination treatment; Dunnett's test). (b) Immunoblots of cell lysates evaluating ErbB and ALK downstream signaling in ALK0413 cells treated with 1000 nM alectinib for 1, 3, 24, and 48 h and 13 days. (c) Cell proliferation assay of ALK0413 cells cultured with alectinib, lorlatinib, dacomitinib, or neratinib, or combinations of alectinib and lorlatinib with 100 nM or 300 nM of dacomitinib or neratinib. (d) ALK0413 cells cultured with alectinib, dacomitinib, or alectinib + dacomitinib (100 nM and 300 nM) were evaluated for caspase-3/7 activity relative to that in vehicle-treated cells. (e) Immunoblots evaluating ErbB signaling and ALK downstream signals from cell lysates of ALK0413 cells treated with 1000 nM alectinib, 100 nM dacomitinib, and their combination for 24 h. (f, g) ALK0413 cells were seeded at 1×10^6 cells in each flask. Cells were treated with vehicle, alectinib (100 nM), dacomitinib (10 nM) and a combination of alectinib + dacomitinib on the following day. The scheme of treatment was shown (f). Cell numbers were measured using a cell counter at Weeks 0, 1, 2, 3, 4, 5. If the cell number was 1×10^4 or less, it is indicated and considered a limitation of the cell counter (n = 1) (g).

Supplementary Figure 4

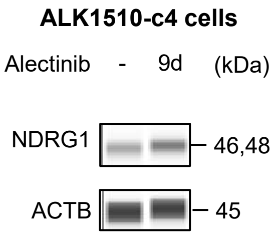

Supplementary Figure 4. NDRG1 expression was increased after 9 days of alectinib exposure.

Immunoblots of cell lysates from 1510-c4 cells treated with 1000 nM alectinib for 9 days.

Supplementary Figure 5

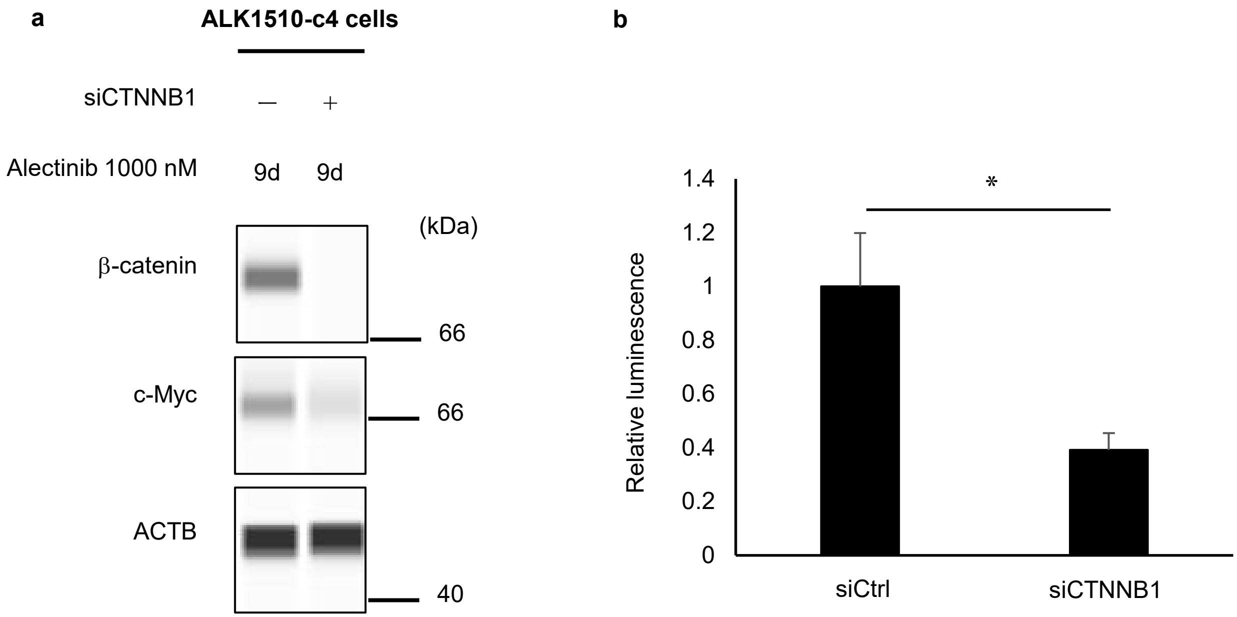

Supplementary Figure 5. Effect of β-catenin knockdown in ALK1510-c4 cells

(a) Immunoblots of cell lysates from ALK1510-c4 cells. Each siCtrl or siCTNNB1 transfected ALK1510-c4 cells were treated with alectinib 1000 nM for 9 days. (b) Relative cell growth levels in ALK1510-c4 cells. Each siCtrl or siCTNNB1 transfected ALK1510-c4 cells were treated with alectinib 1000 nM for 9 days. (mean [SD] of n = 3 experiments, *$P < 0.05$ between siCtrl and siβ-catenin treatment; Student's *t*-test).

Supplementary Figure 6

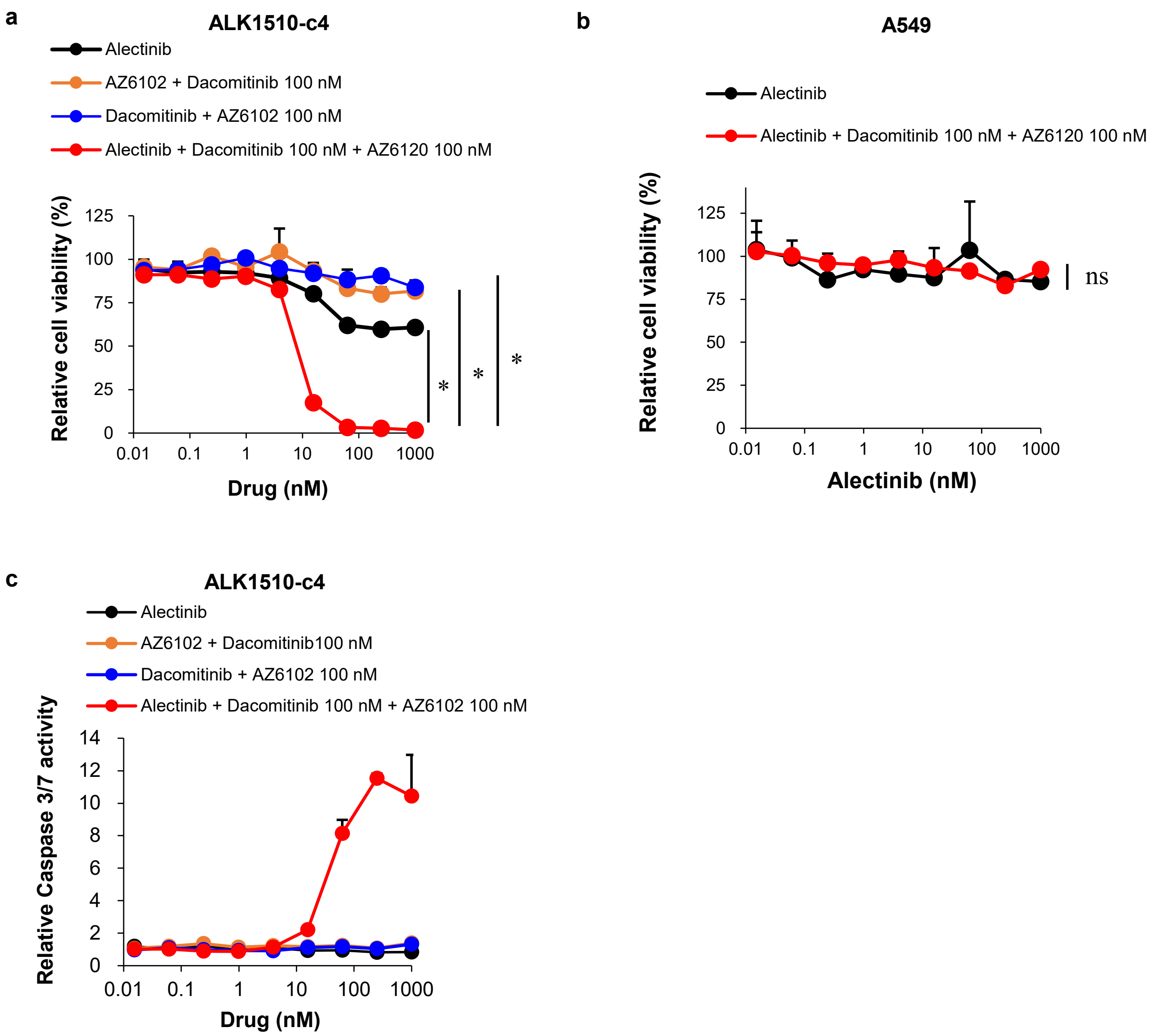

Supplementary Figure 6. Sensitivity of AZ6102 and dacomitinib

(a) Cell proliferation assay of ALK1510-c4 cells cultured with alectinib or AZ6102 + dacomitinib 100 nM or dacomitinib + AZ6102 100 nM or triple combination of alectinib + dacomitinib 100nM + AZ6102 100 nM (mean [SD] of $n = 3$ experiments, *$P < 0.05$ triple combination treatment vs alectinib, double combination treatment; Dunnett's test). (b) Cell proliferation assay of A549 cells cultured with alectinib or triple combination of alectinib + dacomitinib + AZ6102 (mean [SD] of $n = 3$ experiments, statistical analysis between alectinib and triple combination treatment was analyzed using Student's *t*-test). (c) Caspase-3/7 activity relative to vehicle treatment was assessed in ALK1510-c4 cell treatment with the drugs described in Fig. S6a (mean [SD] of $n = 3$ experiments).

Supplementary Figure 7

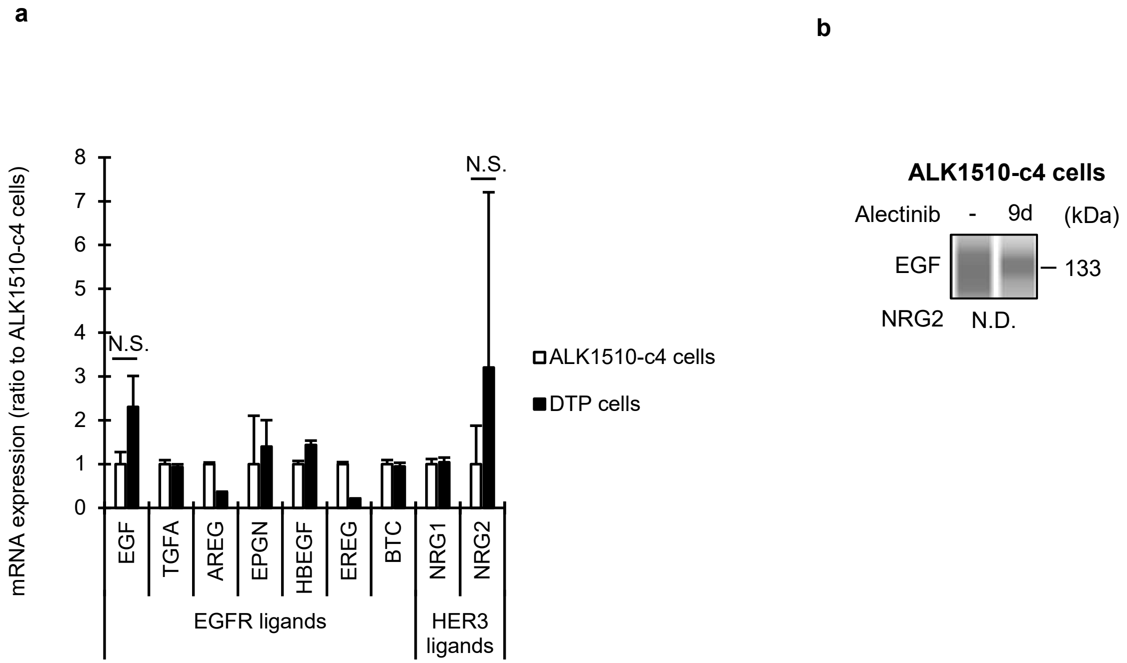

Supplementary Figure 7. Expression levels of EGFR ligands were analyzed.

(a) Levels of mRNA expression of ligands of EGFR and HER3 in DTP cells relative to levels in 1510-c4 cells was analyzed by RNA-seq. **P* < 0.05 between 1510-c4 cells and DTP cells in the Student's *t*-test by Holm method. N.S. means not significant. (b) Immunoblots of cell lysates from 1510-c4 cells treated with 1000 nM alectinib for 9 days. ACTB for this sample was identical to supplementary Figure. 4

Supplementary Figure 8

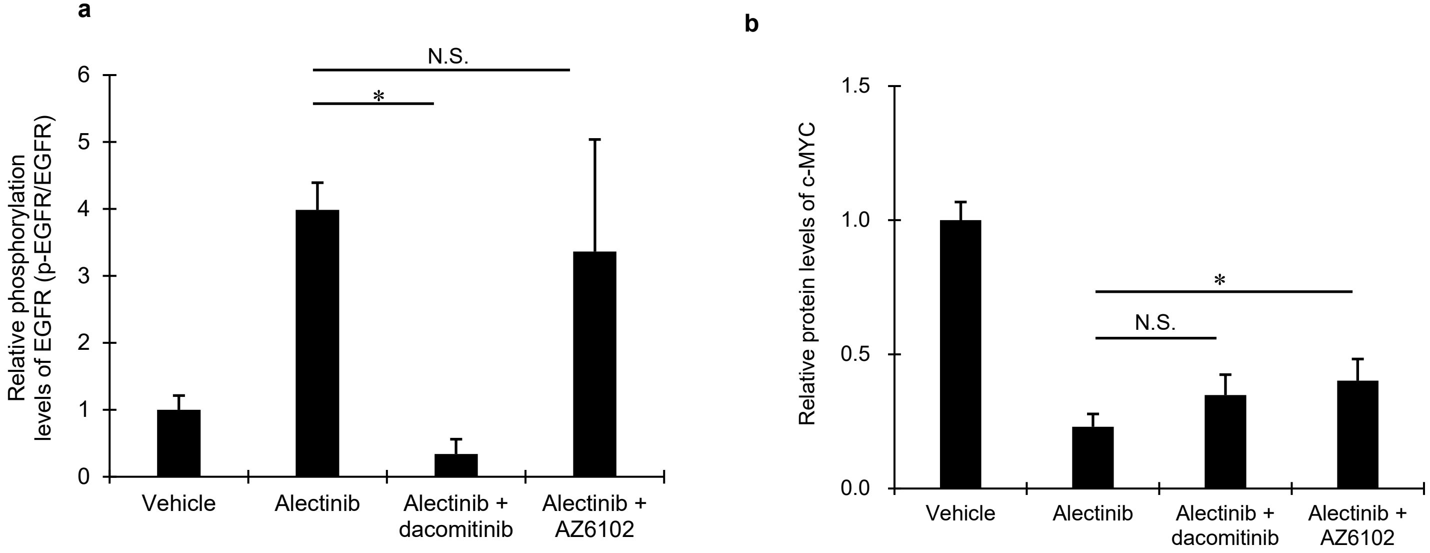

Supplementary Figure 8. Levels of EGFR phosphorylation and c-Myc protein were analyzed.

(a, b) Phosphorylation levels of EGFR (Y1068) per EGFR protein (a), and c-MYC protein levels (b) were measured in DTP cells treated with alectinib (1000 nM) alone, alectinib + dacomitinib (100 nM), or alectinib + AZ6102 (100 nM) for 9 days, relative to vehicle-treated ALK1510-c4 cells. Measurements were performed using ELISA. *P < 0.05 between alectinib alone and combination treatment; Dunnett's test. N.S. means not significant.

Supplementary Table

Cell line	EML4-ALK	Others
ALK1510	variant 1	mTOR A2210P<br>TP53 Q38*
ALK0413	variant 2	IDH2 R140Q

Supplementary Table. Mutation status of patient-derived ALK-positive cell lines.

The mutation status of two ALK-positive patient-derived cell lines is shown. Total nucleic acid was extracted from ALK1510 and ALK0413 cells, and mutations were detected by Oncomine™ Pan-Cancer Cell-Free Assay.

Whole western blots of Fig 1b

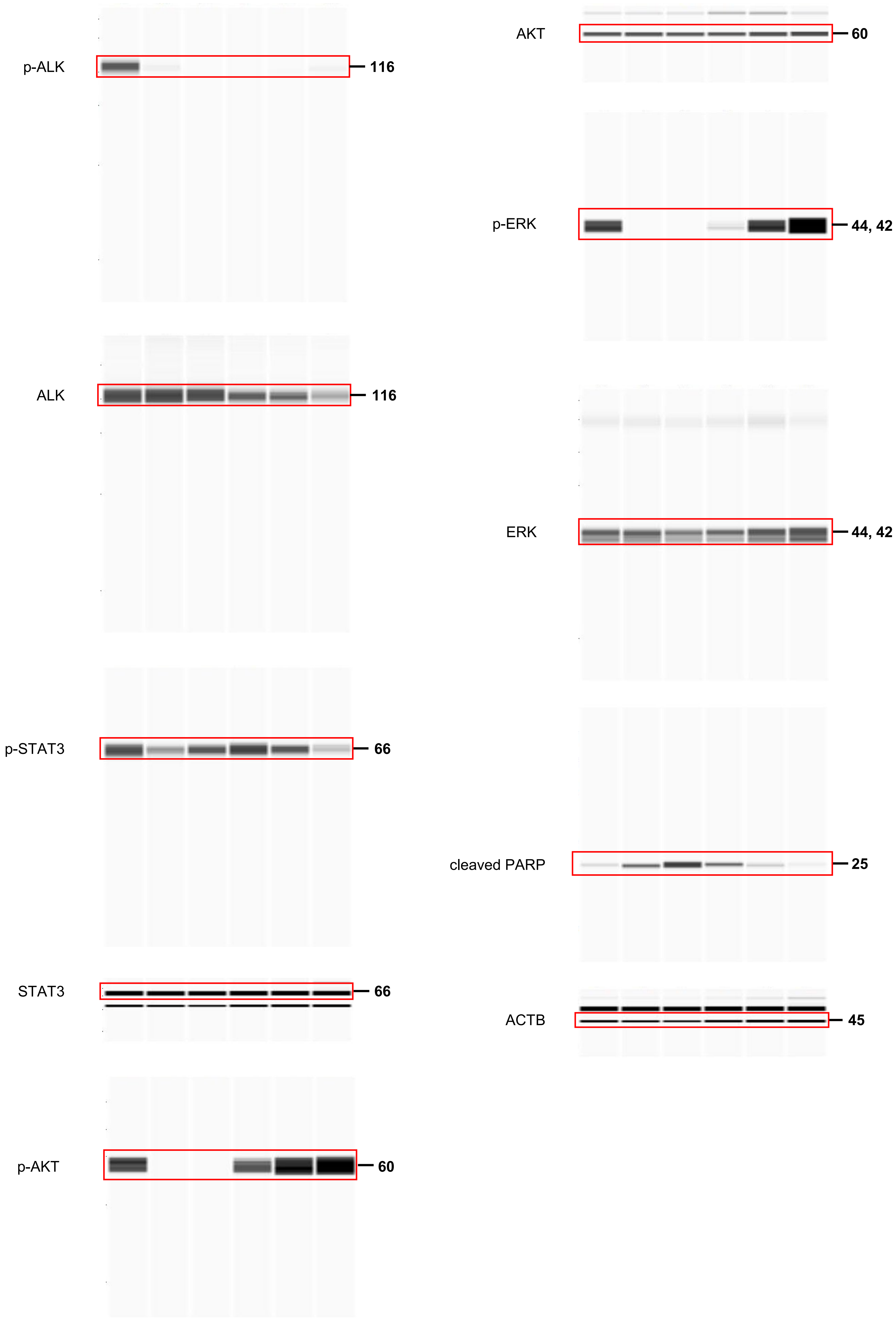

Whole western blots of Fig 1c

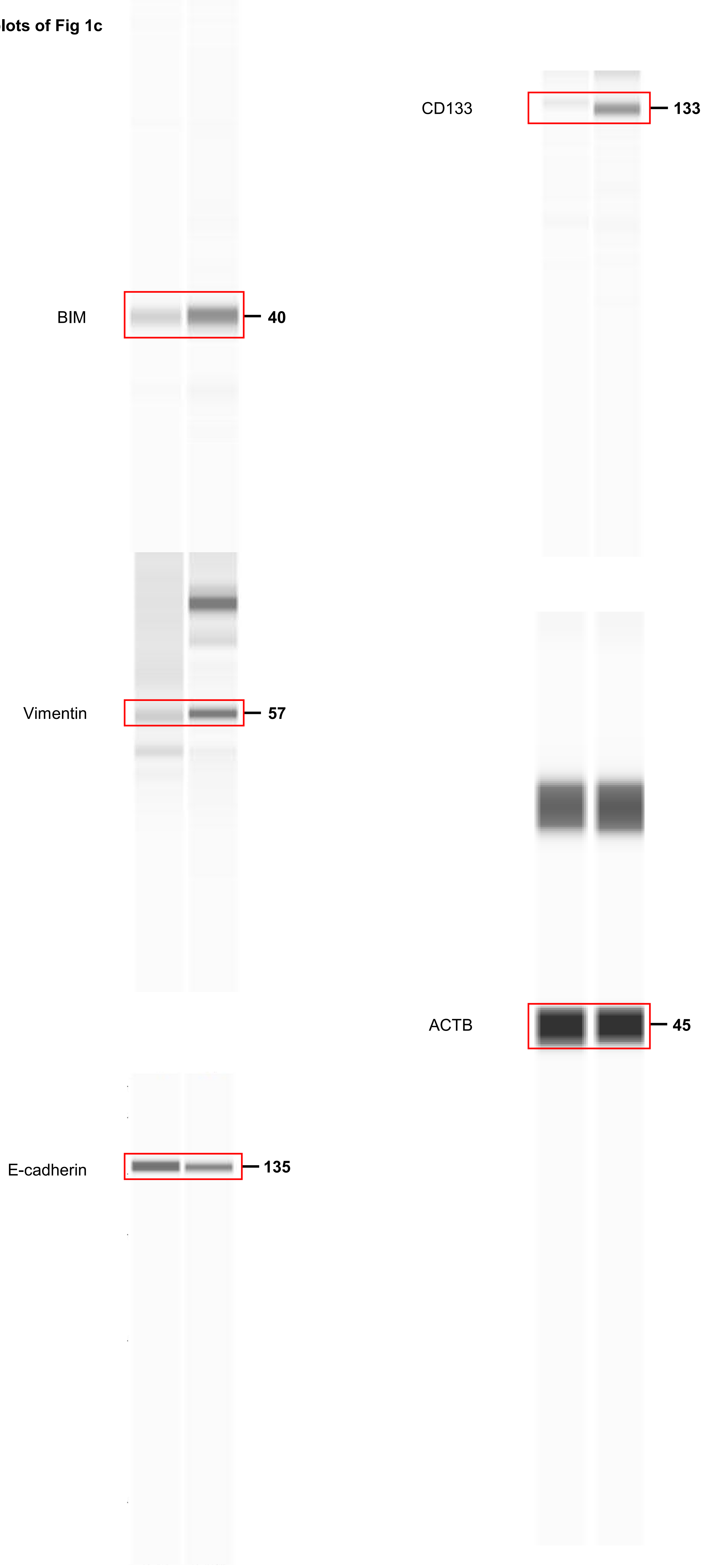

Whole western blots of Fig 1d

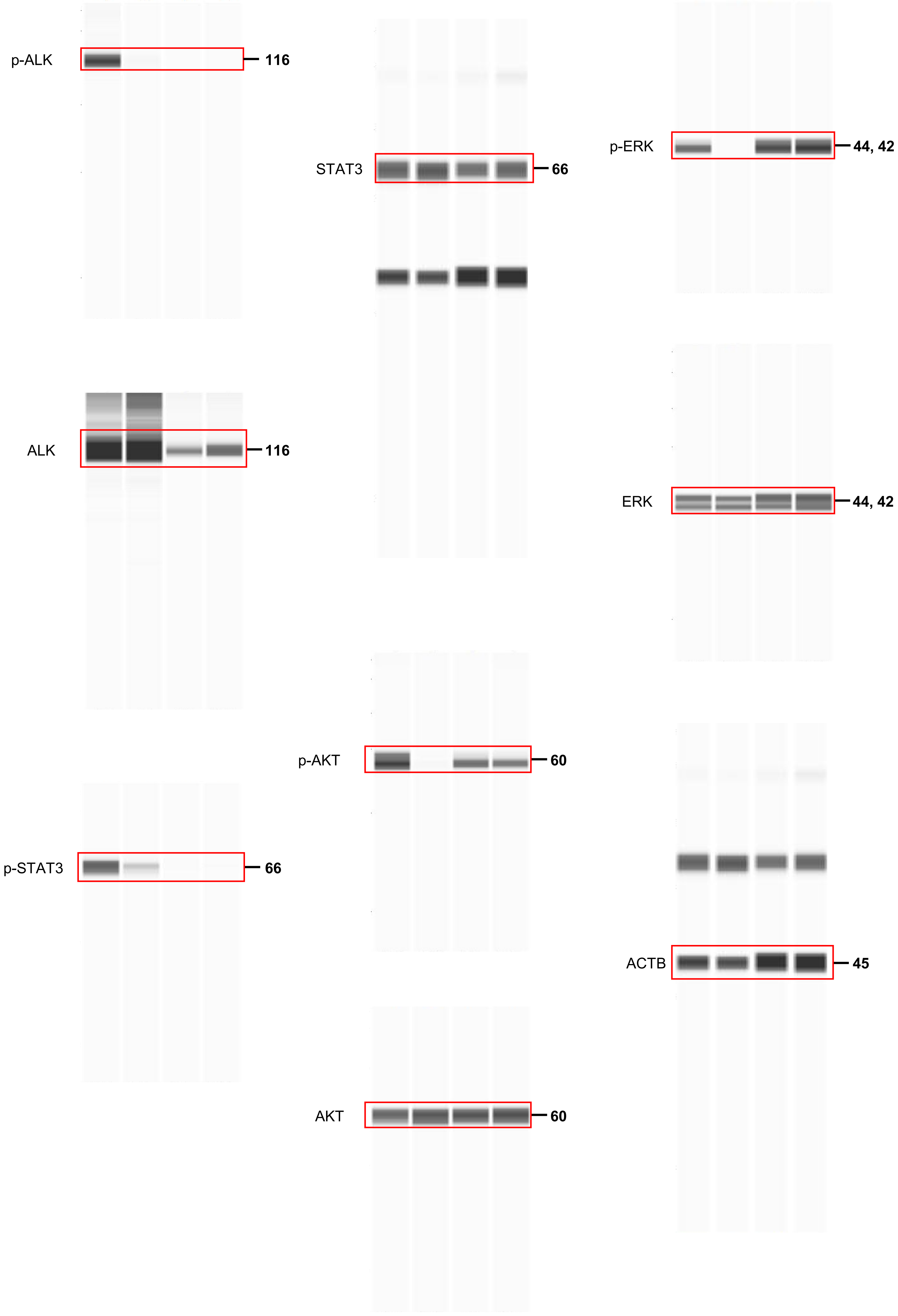

Whole western blots of Fig 1g

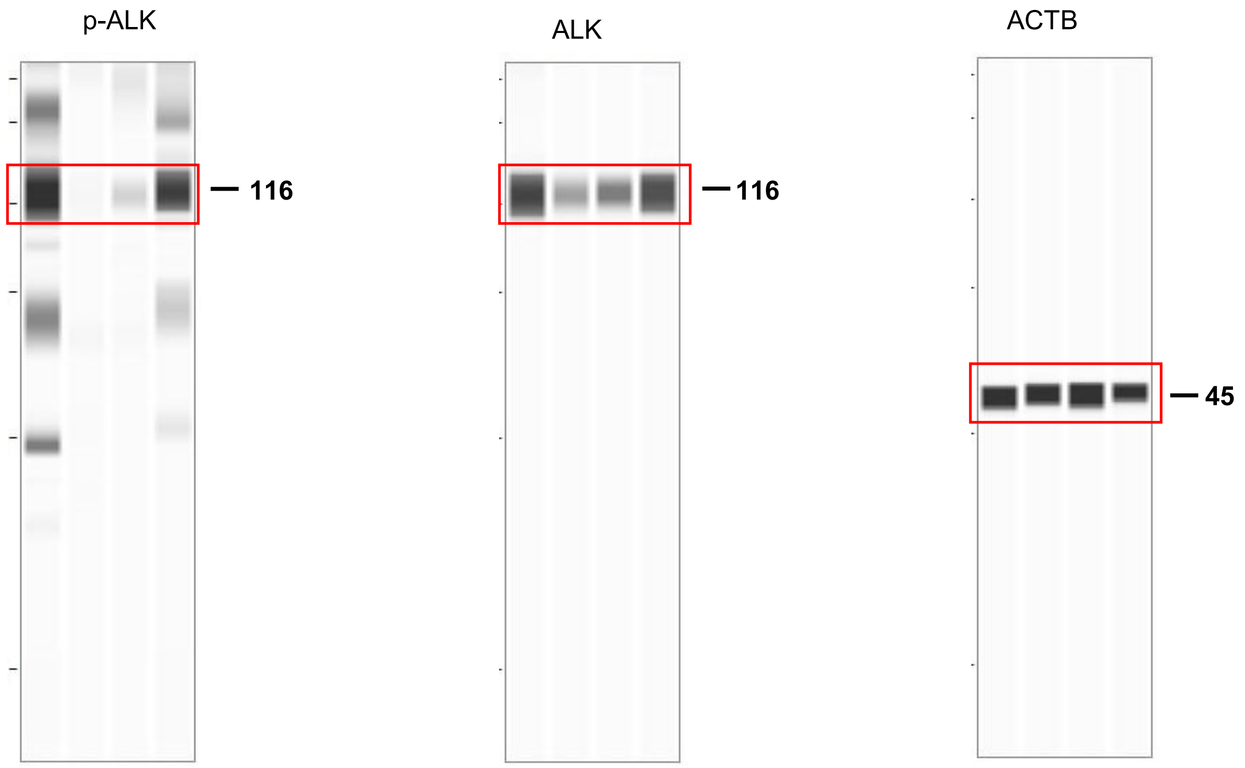

Whole western blots of Fig 3a

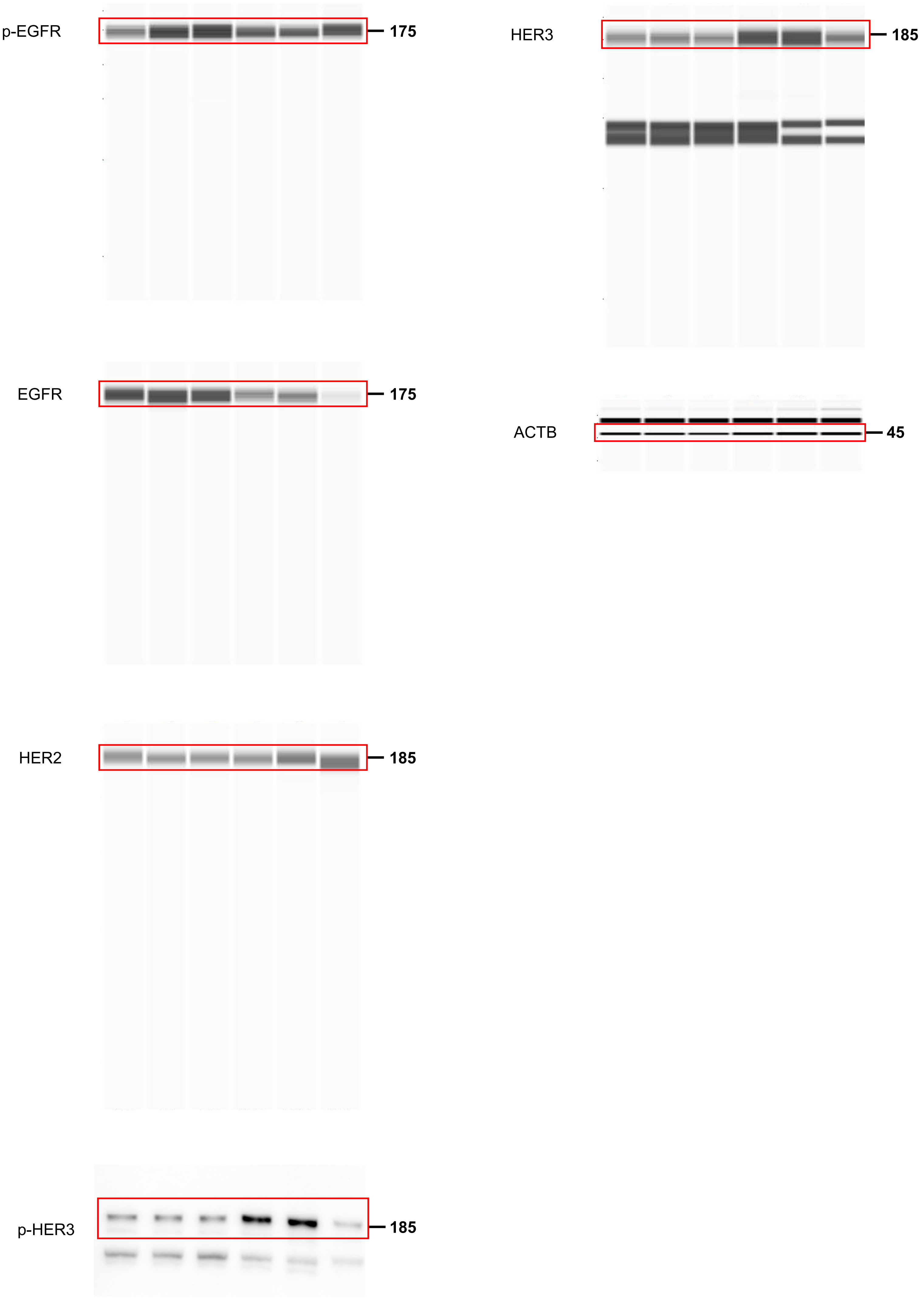

Whole western blots of Fig 3d

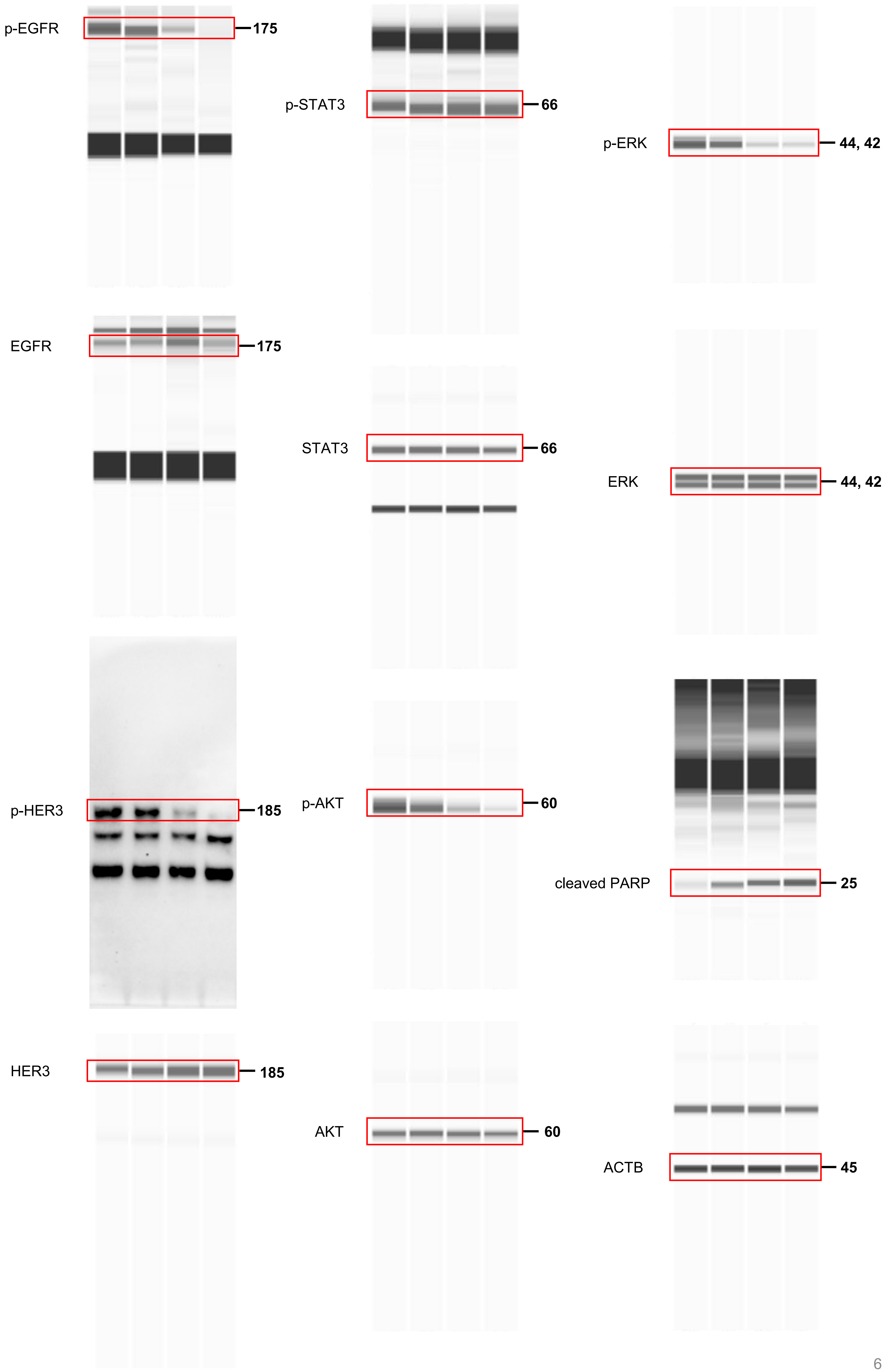

Whole western blots of Fig 4c

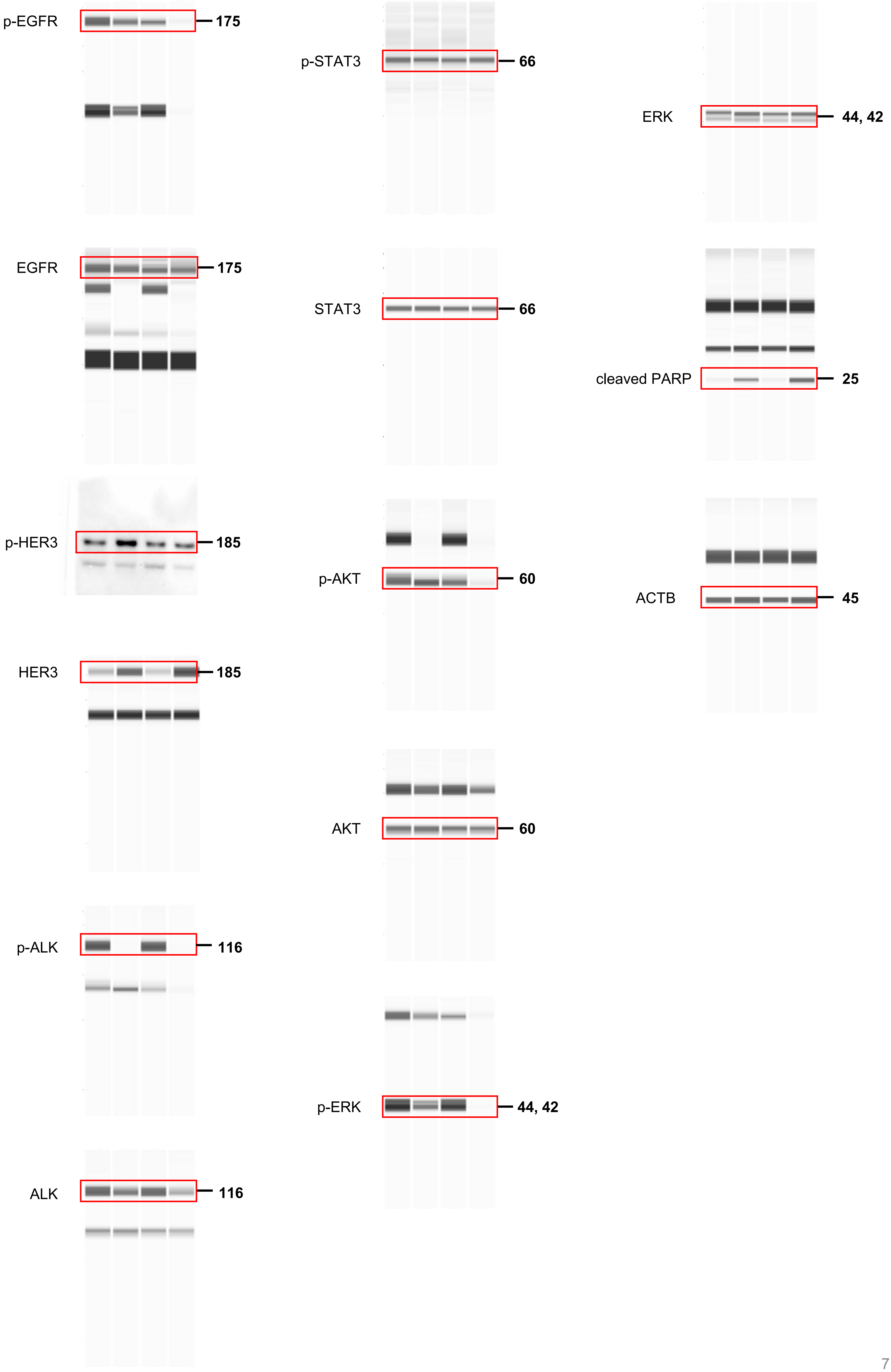

Whole western blots of Fig 4e

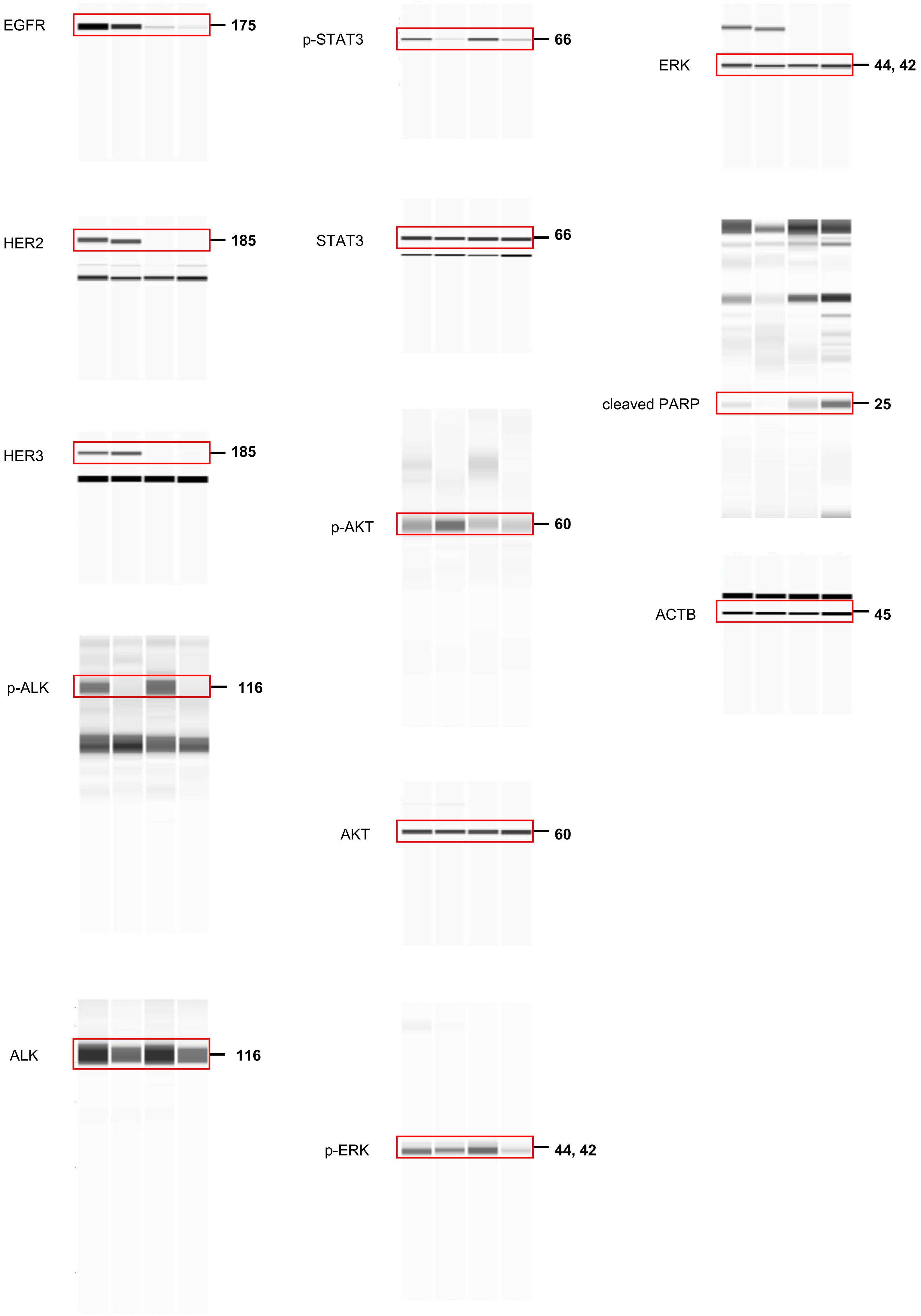

Whole western blots of Fig 4g

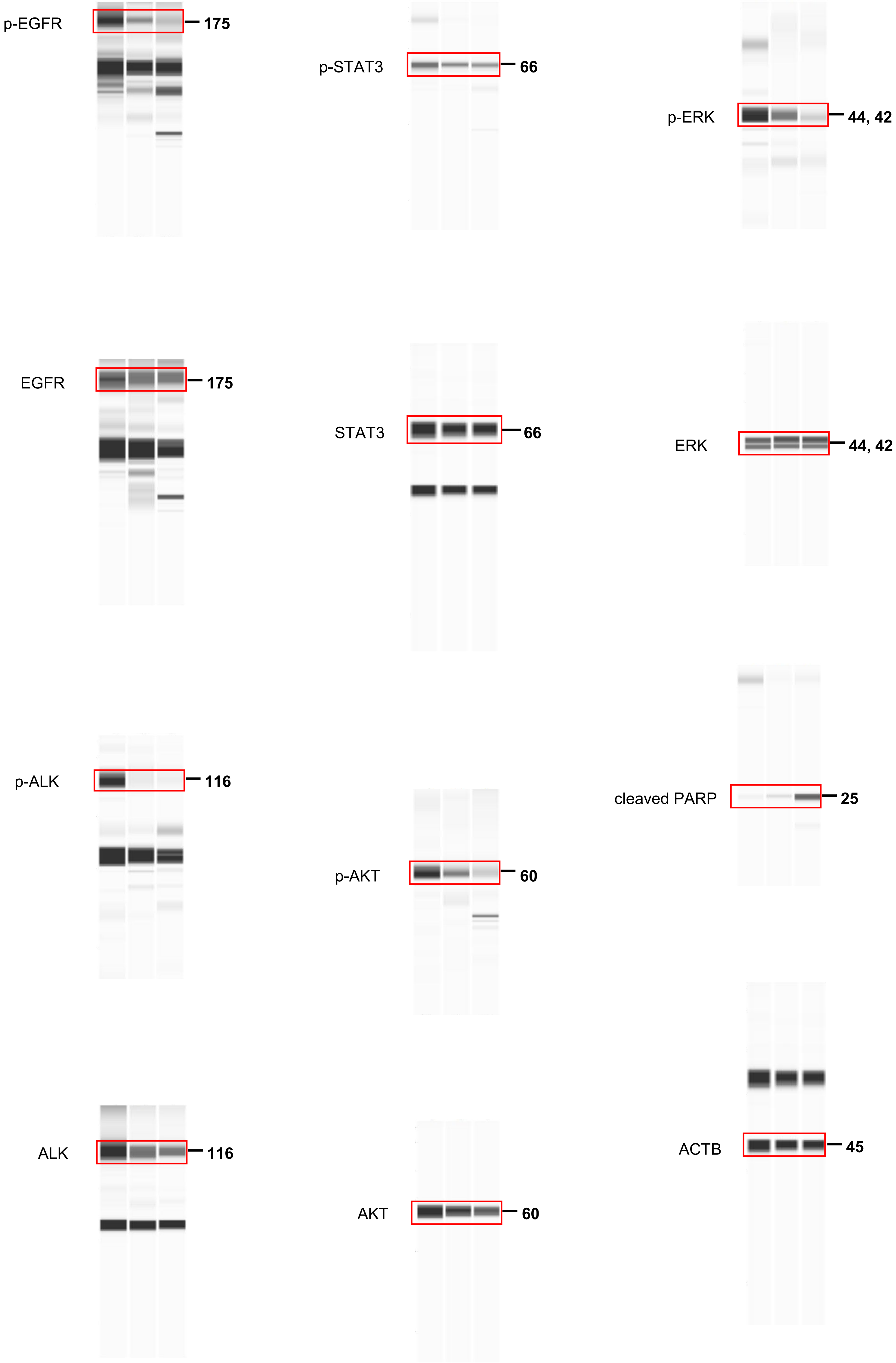

Whole western blots of Fig 5b

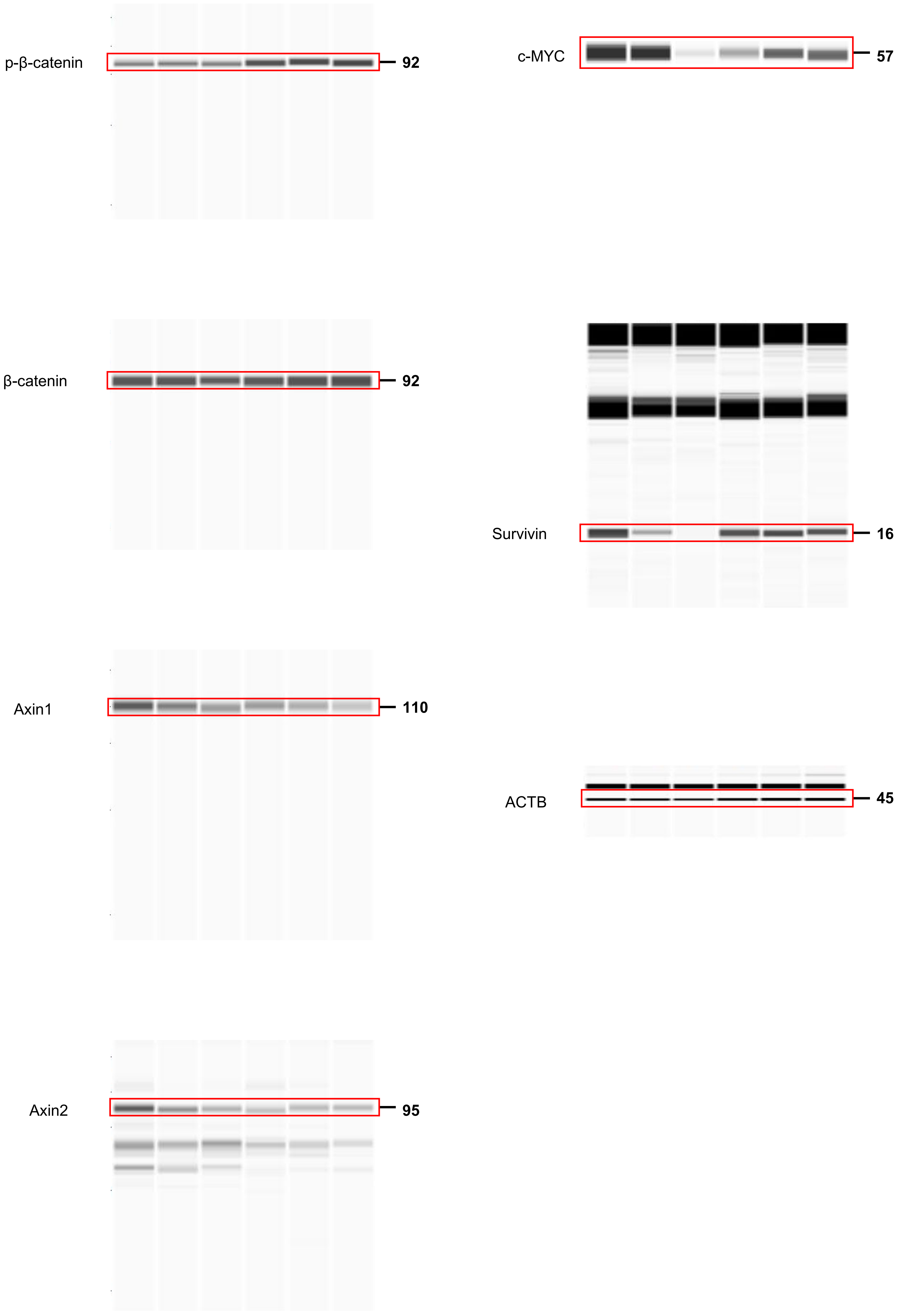

Whole western blots of Fig 5c

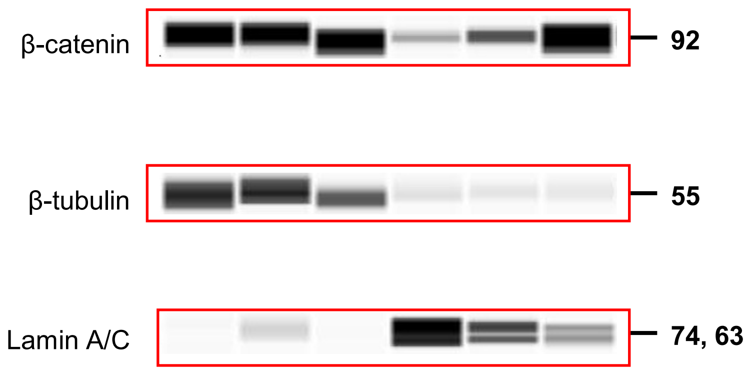

Whole western blots of Fig 6d

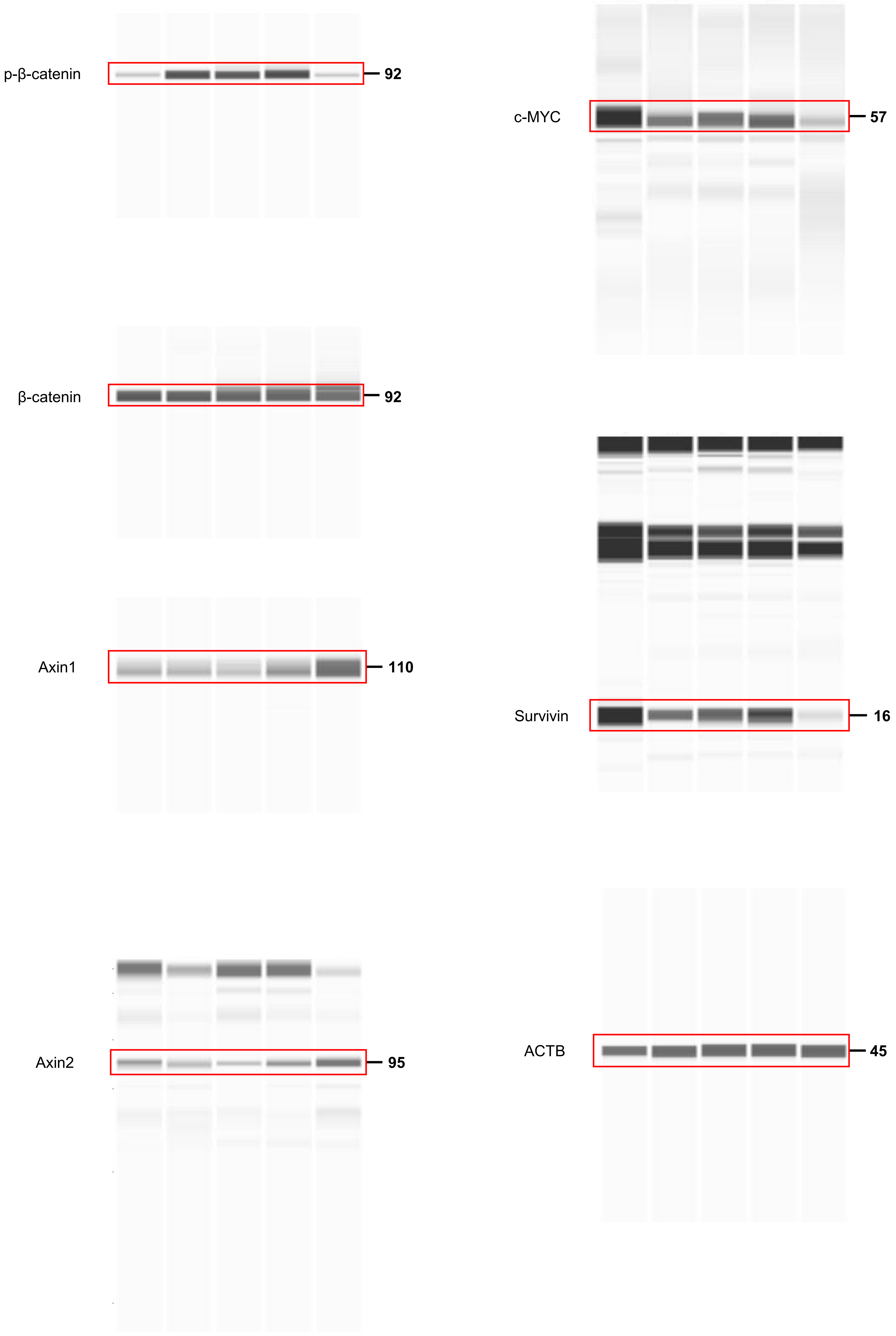

Whole western blots of Fig 7b

p-β-catenin
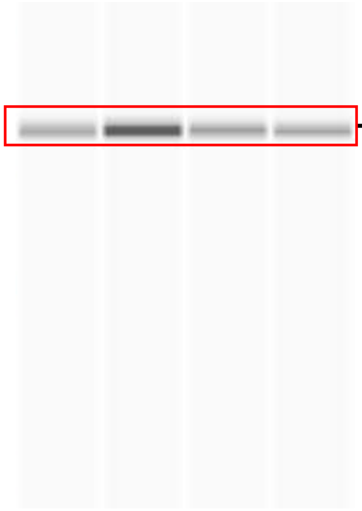
92

β-catenin
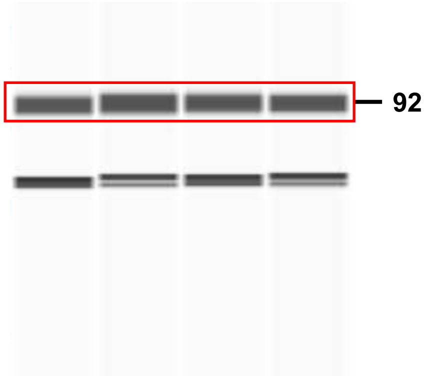
92

Axin1
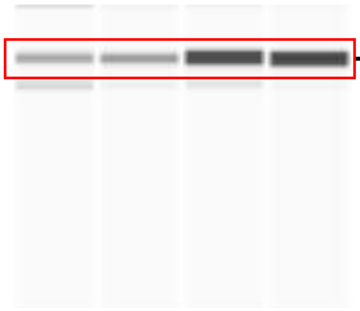
110

Axin2
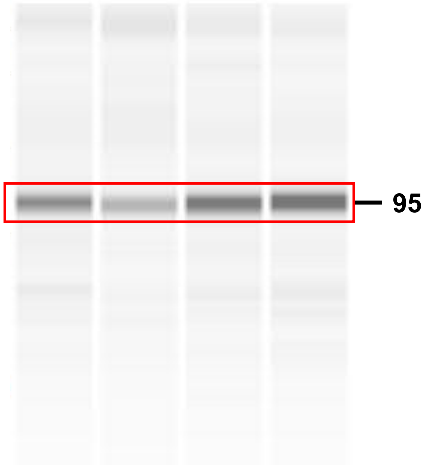
95

c-MYC
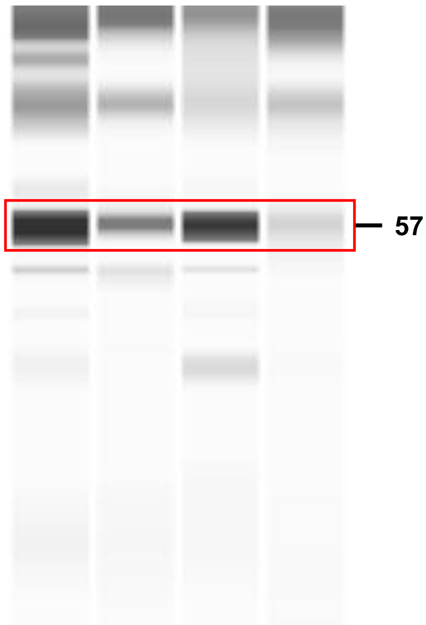
57

Survivin
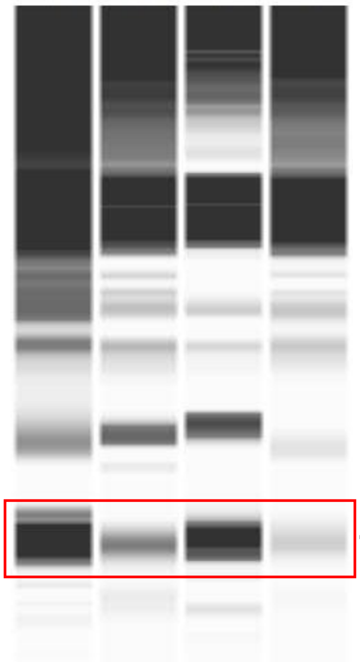
16

ACTB
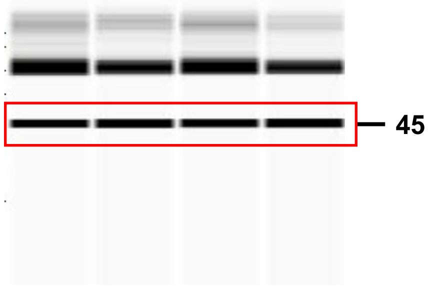
45

Whole western blots of Fig 7e

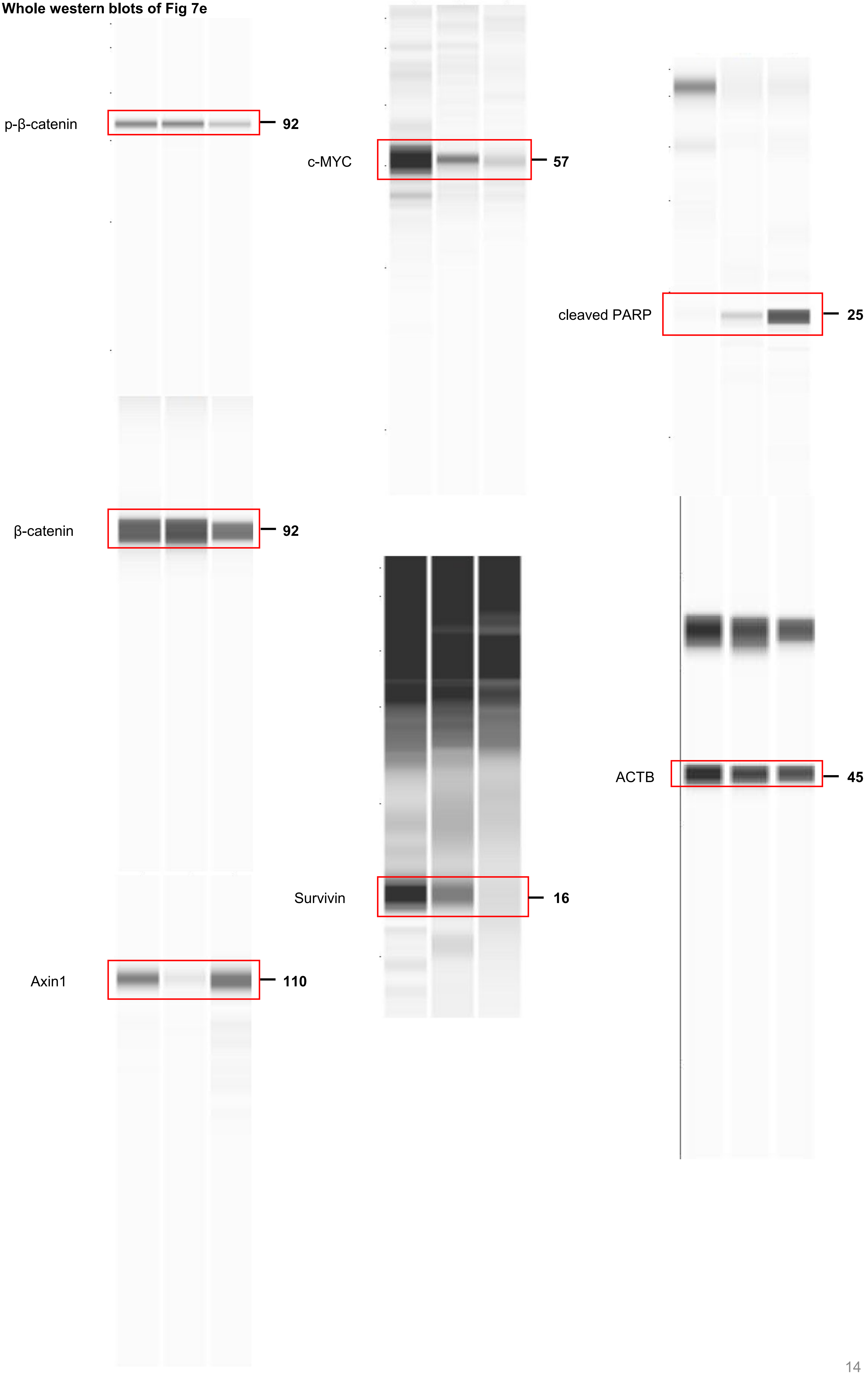

Whole western blots of Fig 8c

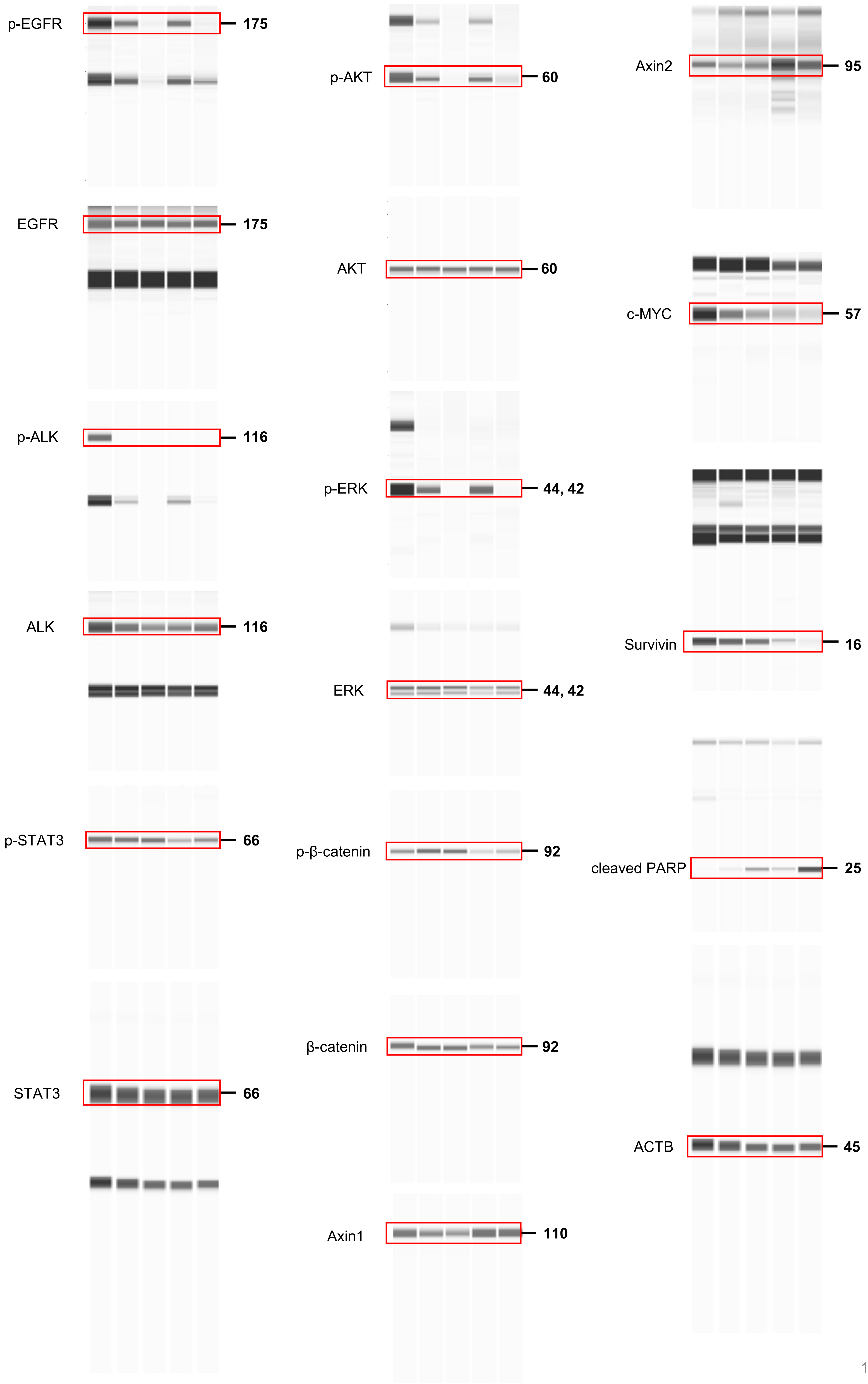

Whole western blots of Fig 8e

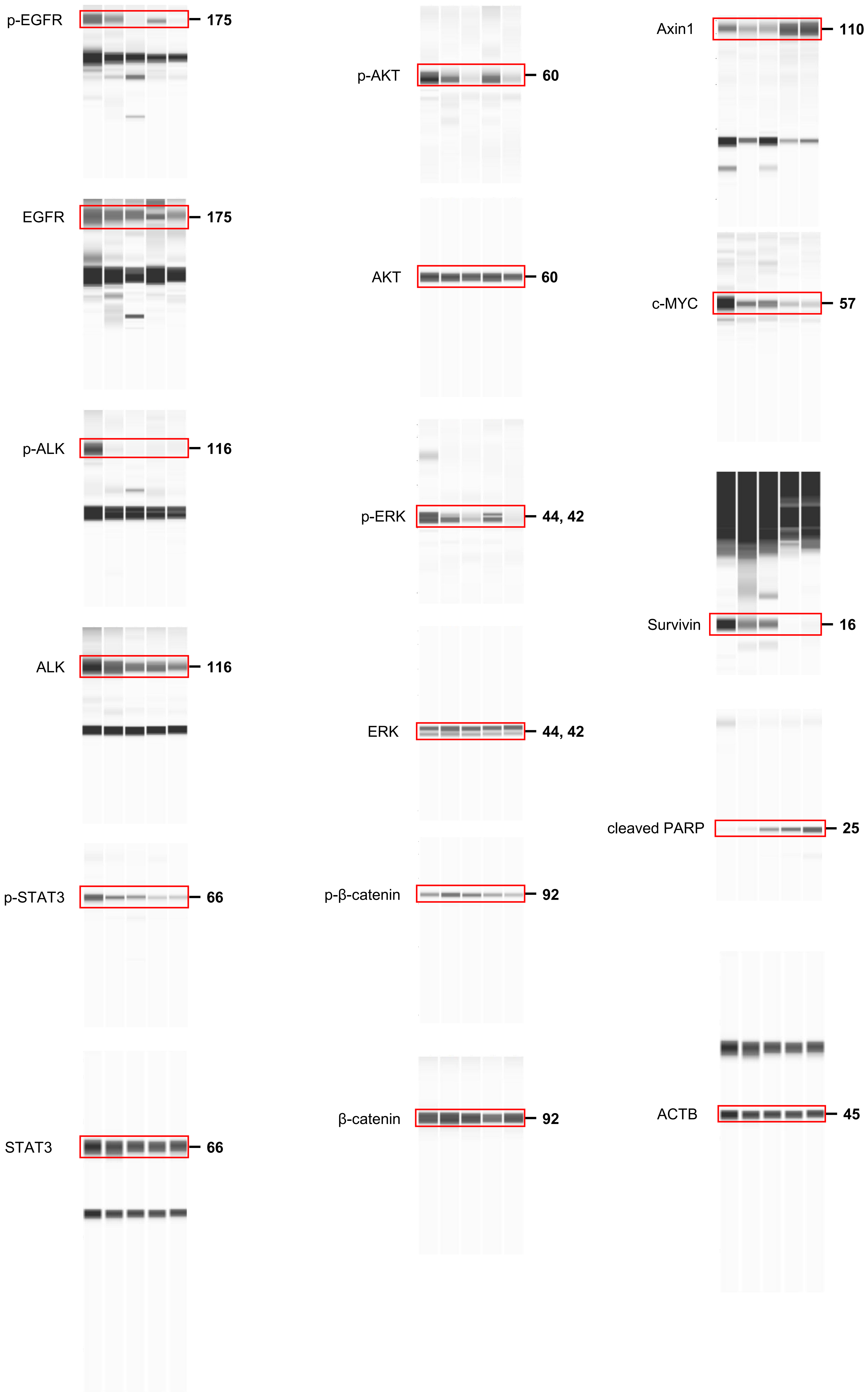

Whole western blots of Supplementary figure 3b

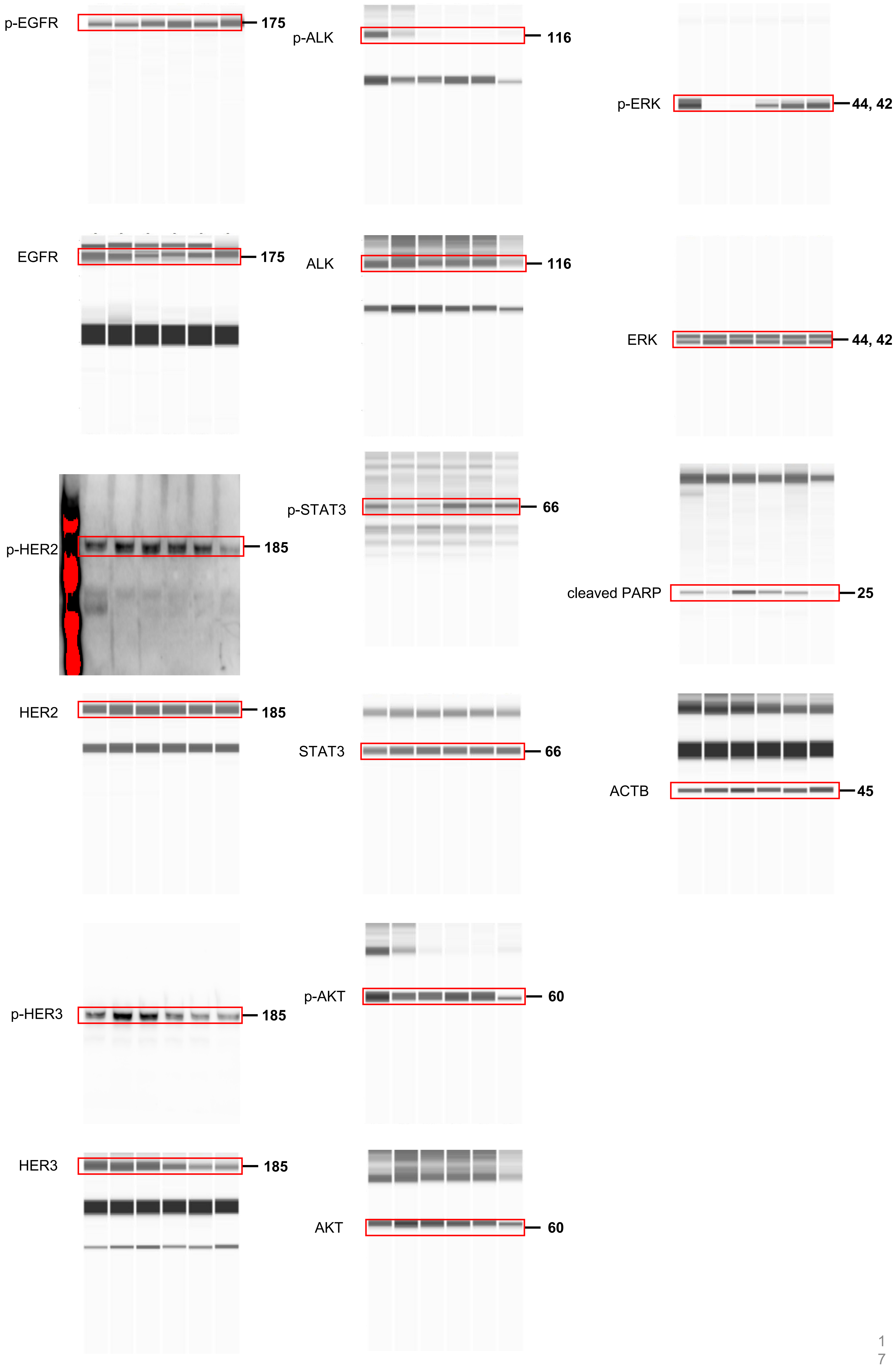

Whole western blots of Supplementary Figure 3e

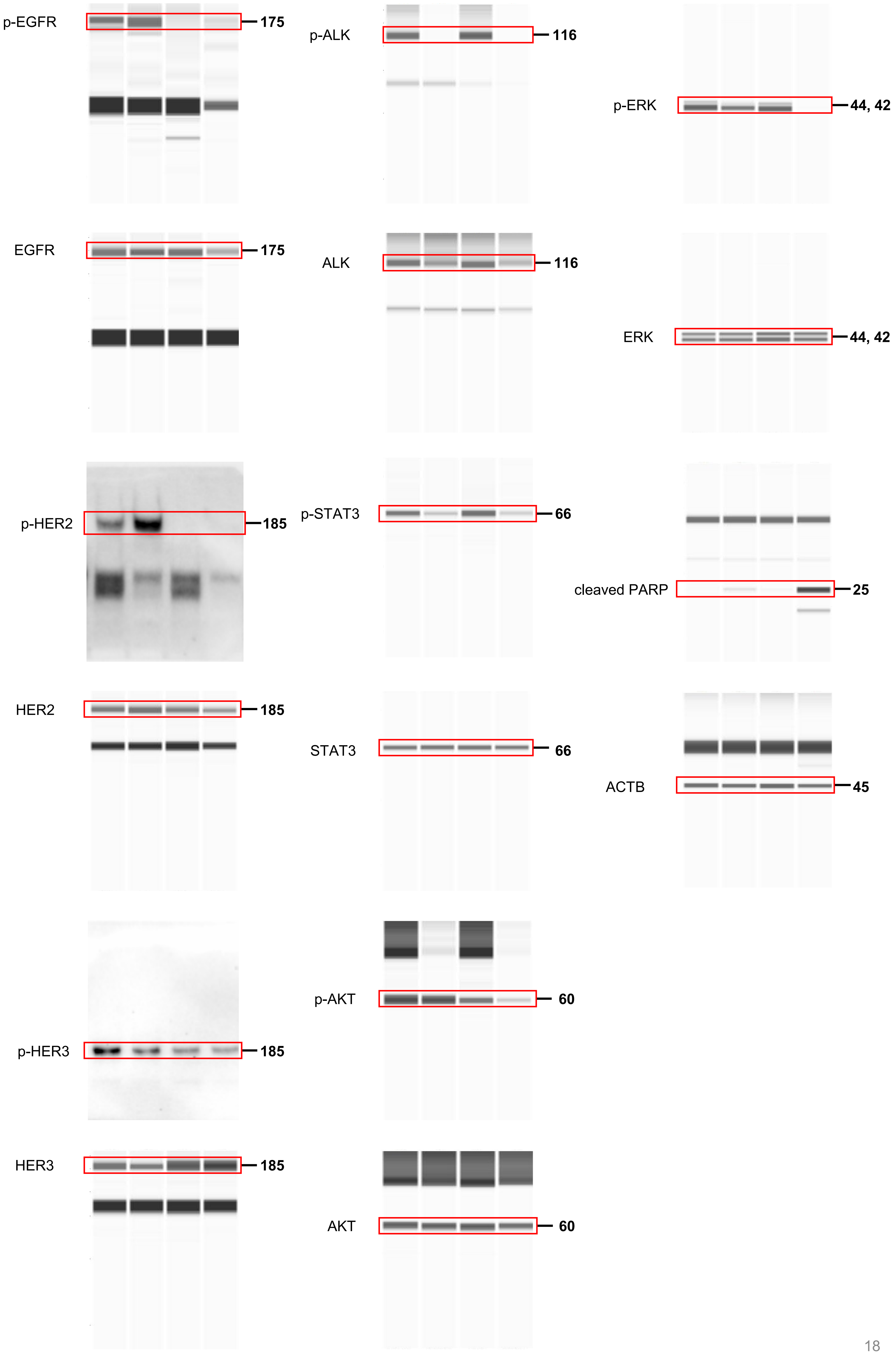

Whole western blots of Supplementary Figure 4

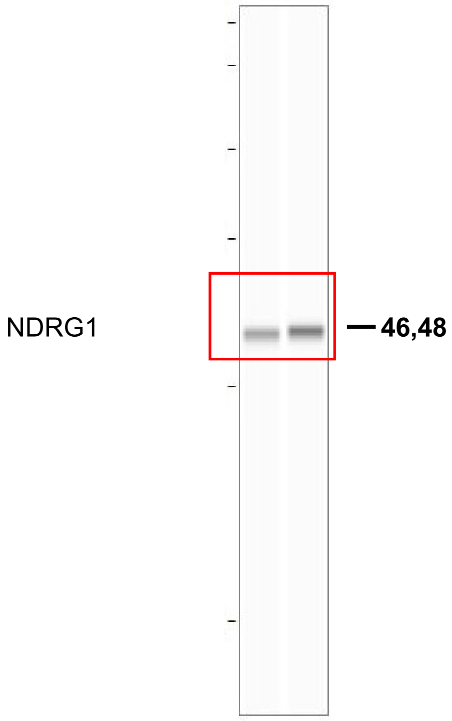

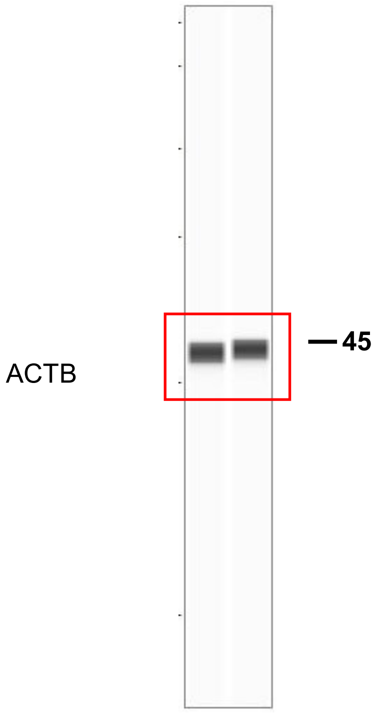

Whole western blots of Supplementary Figure 5a

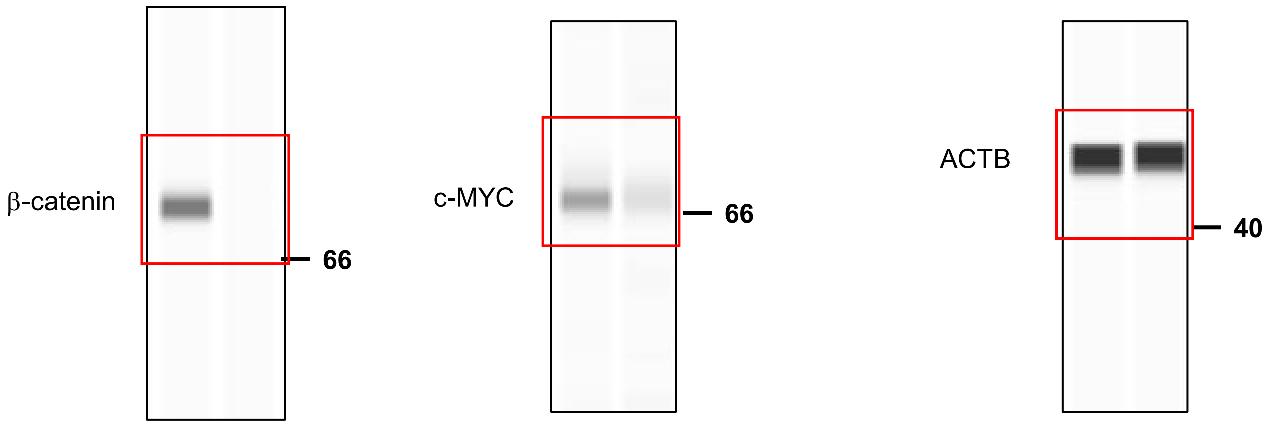

Whole western blots of Supplementary Figure 7b

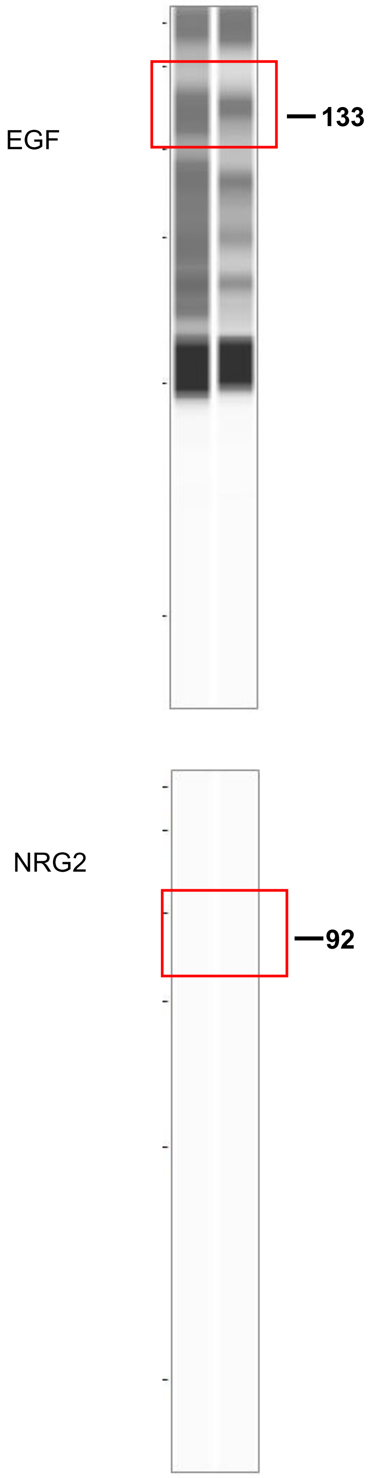

Supplement: Supplementary file 1 — supplementary file [file 41698_2024_757_MOESM1_ESM.pdf]
